# Histotripsy: the first noninvasive, non-ionizing, non-thermal ablation technique based on ultrasound

**DOI:** 10.1080/02656736.2021.1905189

**Published:** 2021

**Authors:** Zhen Xu, Timothy L. Hall, Eli Vlaisavljevich, Fred T. Lee

**Affiliations:** a Department of Biomedical Engineering, University of Michigan, Ann Arbor, MI, USA; b Department of Biomedical Engineering and Mechanics, Virginia Polytechnic Institute and State University, Blacksburg, VA, USA; c Departments of Radiology, Biomedical Engineering, and Urology, University of Wisconsin, Madison, WI, USA

**Keywords:** High intensity ultrasound, ultrasound, physics, imaging, immunotherapy

## Abstract

Histotripsy is the first noninvasive, non-ionizing, and non-thermal ablation technology guided by real-time imaging. Using focused ultrasound delivered from outside the body, histotripsy mechanically destroys tissue through cavitation, rendering the target into acellular debris. The material in the histotripsy ablation zone is absorbed by the body within 1–2 months, leaving a minimal remnant scar. Histotripsy has also been shown to stimulate an immune response and induce abscopal effects in animal models, which may have positive implications for future cancer treatment. Histotripsy has been investigated for a wide range of applications in preclinical studies, including the treatment of cancer, neurological diseases, and cardiovascular diseases. Three human clinical trials have been undertaken using histotripsy for the treatment of benign prostatic hyperplasia, liver cancer, and calcified valve stenosis. This review provides a comprehensive overview of histotripsy covering the origin, mechanism, bioeffects, parameters, instruments, and the latest results on preclinical and human studies.

## Introduction

An important recent trend in medical interventions is a comprehensive drive toward less invasive yet effective procedures. Many diseases can now be addressed using minimally-invasive or noninvasive approaches, and many of these are performed under increasingly sophisticated image guidance. The progression from planar radiation therapy to stereotactic body radiation therapy (SBRT) is one such example, but toxicity still limits treatment volumes and locations [1,2]. Thermal-based ablations are most frequently delivered percutaneously under image guidance and include radiofrequency ablation [3], microwave ablation [4], and cryoablation [5]. These technologies either heat or freeze targeted tissue to produce necrosis. Limitations of thermal modalities include the heat sink effect caused by blood flow which limits the ablation zone and results in a lack of predictability of margins as well as a critical reliance on physician expertise [6-8]. Thermal spread limits treatment of tumors near sensitive structures. High intensity focused ultrasound (HIFU) is a noninvasive thermal ablation technique that uses externally applied ultrasound energy to cause thermal necrosis [9,10]. HIFU has been used clinically to treat uterine fibroids, neurological diseases, and tumors in the prostate, breast, liver, and pancreas, but its clinical use is still infrequent due to anatomic challenges and long procedure times [11-13].

Histotripsy is a noninvasive focused ultrasound technology similar to HIFU [14-17]. However, the underlying mechanism of histotripsy is fundamentally different, relying instead on a mechanical effect at the cellular level to destroy tissue. The term ‘histotripsy’ was coined at the University of Michigan in 2004 [14]. In Greek, ‘Histo’ means ‘soft tissue’, and ‘tripsy’ refers to breakdown. In contrast to thermal HIFU which uses continuous or long bursts of ultrasound at moderately high intensity and high duty cycle (ultrasound on-time/total treatment time ≥10%) to heat tissue [18], histotripsy uses short ultrasound bursts (microseconds in length) with a low duty cycle (≤1%) to minimize heating [19], and higher peak pressure amplitudes to generate acoustic cavitation from endogenous gas in tissues. Acoustic cavitation is the generation, oscillation, and collapse of microbubbles activated by ultrasound [20]. Very high ultrasound pressure causes inertial expansion and collapse of cavitation bubbles that impart localized intense strain that can fracture cells [21] into an acellular debris [16]. Ultrasound imaging can be used to guide and monitor the histotripsy procedure in real-time. In contrast to many existing minimally-invasive techniques, histotripsy can noninvasively remove tissue. When histotripsy is applied to a tissue-fluid interface (e.g., blood clots or cardiac tissue), the tissue is eroded from the surface inwards creating perforations with sharp boundaries [14,17]. When targeting histotripsy inside a bulk tissue (e.g., a tumor), histotripsy liquifies the target tissue to an acellular homogenate, and the debris is absorbed over 1–2 months by the body, leaving small scars [22,23].

Prior to the invention of histotripsy, cavitation created during thermal HIFU was known to cause structural mechanical damage to tissue [24,25]. Soft tissue cavitation damage was also observed during lithotripsy, particularly when using high pulse repetition frequencies [26,27]. The effect was further explored as an intentional surgical technique termed ‘shock wave therapy’ targeting soft tissue and tumor cells [28-30]. Follow-up work developed a more controllable and likely more durable piezoelectric sound source [31,32]. Prior studies [27,29,32-36] show that shockwave therapy was able to intentionally produce mechanical tissue disruption in the kidney, liver, and thrombus in animal models, consistent with what later became known as histotripsy. The negative phase of the acoustic wave following the positive shock for these systems is much longer (several microseconds) than current histotripsy systems, creating a larger focal zone with more sparse cavitation sites, though complete liquefaction was possible with a sufficiently large number of pulses.

The ability to effectively remove tissue allows histotripsy to be used in applications that are not possible with thermal techniques. The non-thermal nature also enables histotripsy to overcome many of the limitations associated with thermal devices (e.g., heat sink effect, lack of precise margins, and predictability). Histotripsy has been investigated for many pre-clinical applications, including treatment for tumors in the liver [37-41], kidney [23,40,42,43], and prostate [44,45], neurological diseases [46,47], thrombosis [48-51], hematoma [52-54], neonatal and fetal congenital heart disease [17,55], valvular diseases [56,57], kidney stones [58], abscesses [59], tendons [60], and biofilms [61-63]. Phase I human trials have been undertaken for histotripsy treatment of benign prostatic hyperplasia [64], liver cancer, and calcified valve stenosis [65], with early results suggesting safety and feasibility in humans. This review provides a comprehensive overview of histotripsy, including the mechanism, bioeffects, parameters, instruments, preclinical and clinical studies, and advantages and limitations compared to related devices.

It should be noted that this review focuses on histotripsy, a technique that uses microsecond pulses to generate inertial cavitation. In Section V, we also briefly discuss boiling histotripsy which is a related technique that uses millisecond ultrasound pulses to generate boiling bubbles to liquefy target tissue.

## Mechanism

The key mechanism that forms the basis of histotripsy is the controllable generation of acoustic cavitation and the interaction between cavitation and tissue, which results in tissue breakdown [66,67]. Simulation and experimental evidence demonstrates that nanometer-scale gas pockets exist in tissue and can function as cavitation nuclei [68,69]. The generation of cavitation during histotripsy is achieved when microsecond length pulses reach negative pressures that exceed an intrinsic threshold and overcome the surface tension of existing nanometer gas pockets. This threshold has been measured in *ex vivo* experiments to be 26–30 MPa for water-based tissues such as blood clots, liver, kidney, heart, brain, spleen, pancreas, as well as blood and water [68]. Prior studies suggest this pressure threshold would correspond to the surface tension of gas pockets of 2–5 nm in diameter [69,70]. The intrinsic threshold only changes slightly with frequency. For example, in one experiment, a small increase of 2–3MPa was observed in the intrinsic threshold when the frequency was increased from 345 kHz to 3 MHz [71]. The intrinsic threshold is notably lower for adipose tissue at 14–17 MPa [68]. Pressure fields only need to exceed the intrinsic threshold for a fraction of a microsecond to reliably produce cavitation.

**Figure 1.**
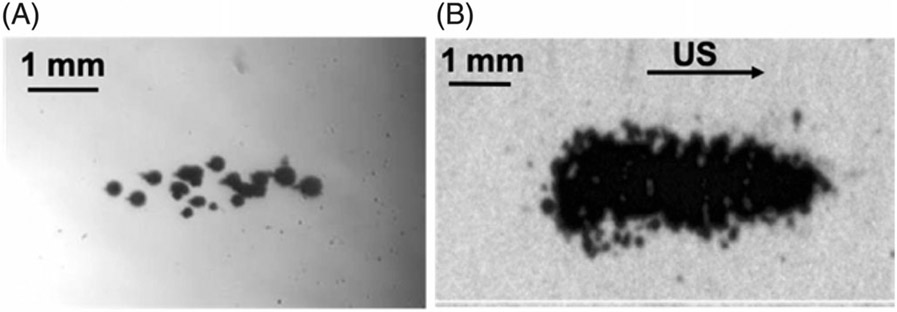
High-speed photograph of cavitation generated by (A) intrinsic threshold histotripsy (modified from ref [69]) and (B) shockscattering histotripsy (modified from ref [73]). Ultrasound propagates from left to right.

Histotripsy typically uses microsecond length ultrasound pulses with a burst duration ≤ 10 acoustic cycles to generate a cluster of microbubbles (cavitation bubble cloud). There are two mechanisms to initiate a cavitation bubble cloud. (1) Intrinsic threshold: When applying an ultrasound pulse of 1–2 cycles in duration with a single high amplitude negative pressure phase, the peak negative pressure directly exceeds the intrinsic threshold, and a bubble cloud is formed as the ultrasound propagates away from the transducer [68] (Figure 1(A)). (2) Shock-scattering: When applying an ultrasound pulse of 3–10 cycles with peak negative pressure below the intrinsic threshold, an initial individual microbubble can be generated probabilistically by the first 1–2 cycles from preexisting cavitation nuclei. At high ultrasound pressures, ultrasound shockwaves with high-frequency harmonic components develop due to non-linear acoustic propagation. Non-linear acoustic propagation compresses the positive phase of the acoustic cycle greatly increasing the peak amplitude. This amplified positive shock-front reflects off the initial microbubble, inverting in phase due to the low impedance of the medium inside the bubble. This results in a strong negative peak traveling back toward the transducer and forming cavitation where the negative pressure exceeds the intrinsic threshold [72]. Using the shock-scattering approach, the bubble cloud is typically fan-shaped and forms back toward the transducer (Figure 1(B)). Using shockscattering, the peak negative pressure of the transmitted acoustic field required to generate cavitation can be as low as 15 MPa.

Cavitation microbubbles are thought to be typically generated in the extracellular matrix. Bubbles grow from 2–5nm to >100 μm followed by energetic collapse within a few hundred microseconds. This rapid expansion and collapse of microbubbles produce very high strain and stress to immediately adjacent cells resulting in a precise mechanical disruption which can occasionally even bisect cells at the ablation boundary [73]. Complete cell disruption within the target does not occur by one pulse and requires the cyclic strain generated by multiple pulses [21].

## Bioeffects

### Inside a bulk tissue

**Figure 2.**
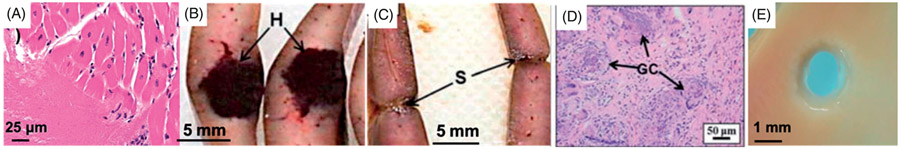
(A) H&E slide of an *ex vivo* porcine cardiac tissue treated by histotripsy shows no intact cells within the treated region and bisected cells at the sharp lesion boundary. (B–C) Gross morphology of the rat liver treated by histotripsy shows a darkly colored hematoma within the histotripsy-treated zone right after treatment (B) and only a small contracted scar on Day 28 (C). (D) Histology of the rat liver treated by histotripsy shows a small focus of residual scar and foreign body giant cells (GC) with calcified undigested debris on Day 28. (B–D) are reproduced based on images from ref [22]. (E) A perforation is generated by histotripsy through a piece of *ex vivo* porcine atrial wall.

When targeting inside a bulk tissue volume, histotripsy can completely disrupt the target tissue achieving a liquid consistency homogenate without intact cells (Figure 2(A)) [74,75]. Transmission Electron Microscopy (TEM) micrographs of tissue treated by histotripsy show little discernable subcellular structure within the treated region [75]. In *in vivo* cases, the acellular debris in the treatment zone is mixed with red blood cells and red blood cell fragments [37]. The acellular debris is absorbed by the body over ~1–2 months, leaving only a small area of fibrous scarring (Figure 2(B-D)) [23].

### At a tissue-fluid interface

When targeting a tissue-fluid interface, histotripsy erodes and removes surface tissue [76]. For example, when targeting at the surface of the atrial septum or a blood clot, histotripsy progressively erodes from the surface, eventually leading to perforation with a sharp boundary through the atrial septum or flow channel through the blood clot (Figure 2(E)) [14,48,49]. The debris particles released after histotripsy are several microns in size or smaller [77].

### Tissue-selectivity

**Figure 3.**
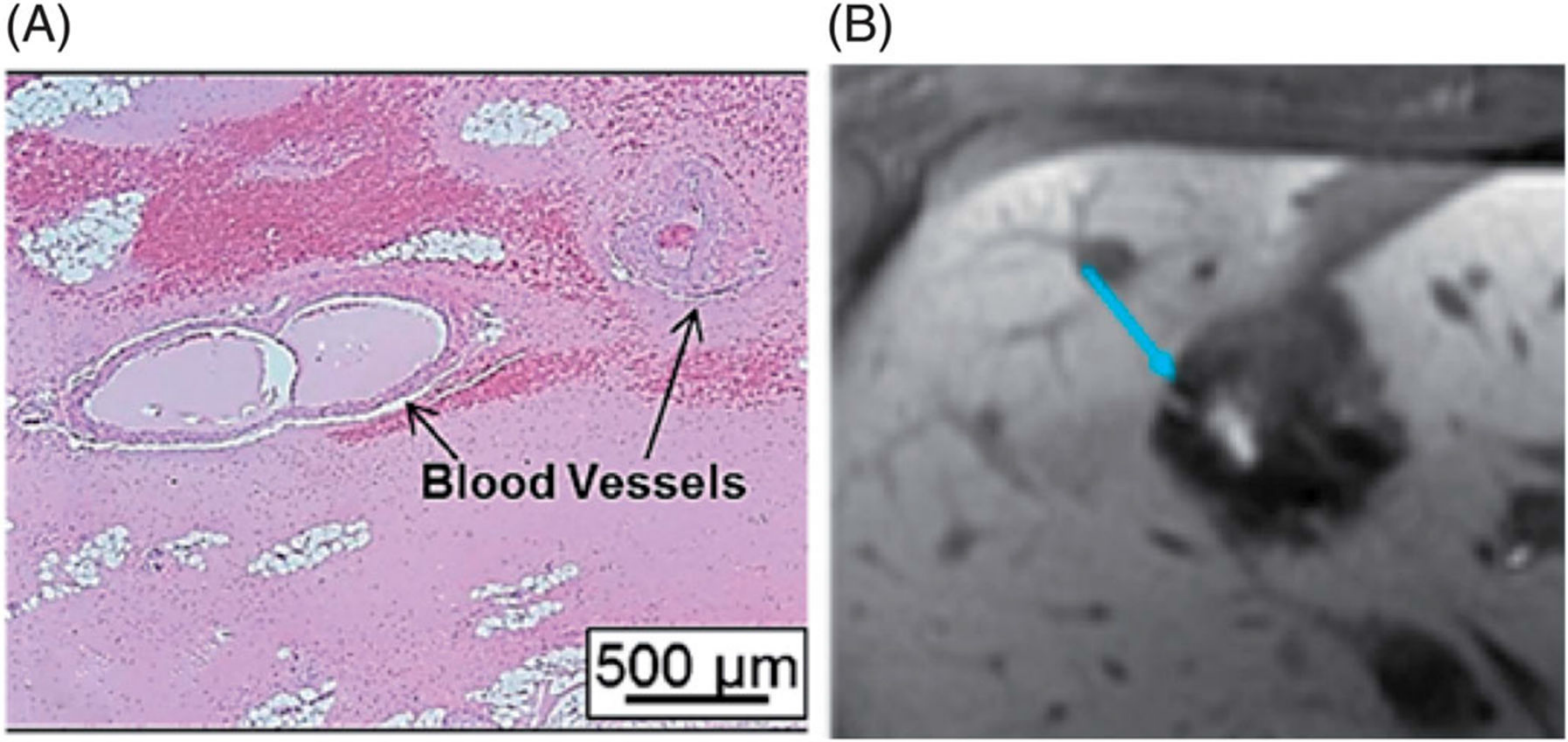
(A) Large blood vessels remain intact and surrounded by acellular debris within the *in vivo* porcine liver treated by histotripsy (reproduced based on an image from ref [37]. (B) Contrast-enhanced MR image showing a patent bile duct (blue arrow) within the dark histotripsy treatment zone (reproduced based on an image from ref [84].

Different tissues have specific resistance thresholds to histotripsy-induced damage, requiring variable numbers of ultrasound pulses and/or ultrasound pressure levels to break down the tissue. Mechanically strong collagen-based tissues (e.g., large vessels, nerves, bile ducts, stroma, or the renal collecting system) have a higher ultimate tensile strength compared to more parenchymal structures (e.g., solid organs and tumors in the liver, kidney, and brain) [78-81]. Also, microbubbles appear constrained to expand to a smaller maximum diameter in stiffer collagen-based tissue compared to the softer cellular tissue, producing a lower strain [80,82,83]. Therefore, it takes a greater number of histotripsy pulses and/or modified pulsing parameters (e.g., lower frequency, higher pressure) to liquefy collagen-based tissue than non-collagenous tissue [69,70,82]. This differential threshold results in a tissue-selective dose-related ablation phenomenon observed in histotripsy [70]. For example, when treating the liver, cells can be completely destroyed, while bile ducts, larger vessels, nerves, and connective tissue within the ablation zone remain intact (Figure 3) [37,84,85]. When treating the kidney, renal cortical tissue can be completely liquefied, while the collecting system remains structurally intact [85,86].

## Ultrasound parameters

The treatment precision of histotripsy is determined by the focal zone size of the ultrasound beam, which in turn depends on the frequency and geometry of the transducer [71,87]. A typical focal zone for a spherical section aperture of 1 MHz is an elliptical shape of 1–2 mm short axis and 2–4 mm long axis. To treat a target volume, multiple focal volumes are stacked together to form the desired shape and size by mechanically or electronically moving the focus across the ablation zone. The individual focal volumes are considered to behave independently, unlike thermal ablation where heat will spread to adjacent regions during a procedure. The effectiveness and extent of histotripsy tissue liquefaction are determined by the dynamics of cavitation bubbles, which depend on the ultrasound parameters used and viscoelasticity of the target tissue [71,88], ultimately determining the number of histotripsy pulses and ultrasound pressure required to produce complete tissue disruption. Histotripsy uses very different parameters from HIFU and boiling histotripsy, as listed in Table 1.

Histotripsy typically has been applied at a frequency of 250 kHz — 6 MHz [71,89], a very high pressure (*p* → 15 MPa), and a pulse duration of ≤10 acoustic cycles [68,72]. Intrinsic threshold histotripsy uses 1–2 cycle pulses, while shock-scattering histotripsy uses 3–10 cycle pulses. The microsecond length of the ultrasound pulse is just long enough to initiate cavitation, but not too long to make the bubble dynamics uncontrollable. A low duty cycle (≤1%) or long time (>1 ms) between microsecond-length pulses prevents heat buildup and allows time for cavitation bubbles to dissolve sufficiently after collapse [90]. Due to the low duty cycle, even with a very high focal pressure and peak intensity, the temporal average intensity used in histotripsy is lower than HIFU. To completely liquefy the target tissue, it takes tens (brain) [47] to hundreds of pulses (liver) [37,40], or a total treatment time of 0.1–60 s per focal location, depending on the mechanical property of the target tissue. As previously described, the number of pulses to achieve complete tissue disruption is greater for stiffer tissue and tissue containing high concentrations of collagen.

## Boiling histotripsy

In 2011, Khokhlova et al. [91] first showed that millisecond length ultrasound pulses can be used to generate boiling bubbles that liquefy the target tissue to acellular debris. Because of the similarity of the mechanism and tissue effects, this process has been termed boiling histotripsy. Boiling histotripsy uses a peak negative pressure (10–20 MPa) lower than histotripsy and higher than HIFU, but with a very high positive shockwave (p+ >70 MPa) [92]. The other parameters used in boiling histotripsy are summarized in Table 1. Tissue atomization has been proposed to be one of the mechanisms by which boiling histotripsy causes tissue damage [93,94]. At the tissue surface, boiling bubbles result in jetting against the tissue surface and fountain projectiles [91,95]. Within a bulk tissue, boiling bubble formation does not always lead to jetting, while recirculation of the fountain projectiles within the treatment volume is necessary for complete tissue homogenization. Boiling histotripsy causes similar bioeffects as histotripsy and has been investigated for the treatment of tumors in the liver [40] and kidney [43] as well as large hematomas [52,54].

## Instrumentation

The key instrumentation components that make up histotripsy systems are a focused ultrasound transducer and an associated electronic driving system. To achieve the high focal pressure required for histotripsy, a large aperture transducer with a low f-number (transducer aperture/focal distance ≤1) [87] and a high focal gain (≥30) is necessary [96,97]. A high voltage pulser that can generate short pulses of thousands of volts and several kW peak power is required to drive the ultrasound transducer to deliver histotripsy.

**Figure 4.**
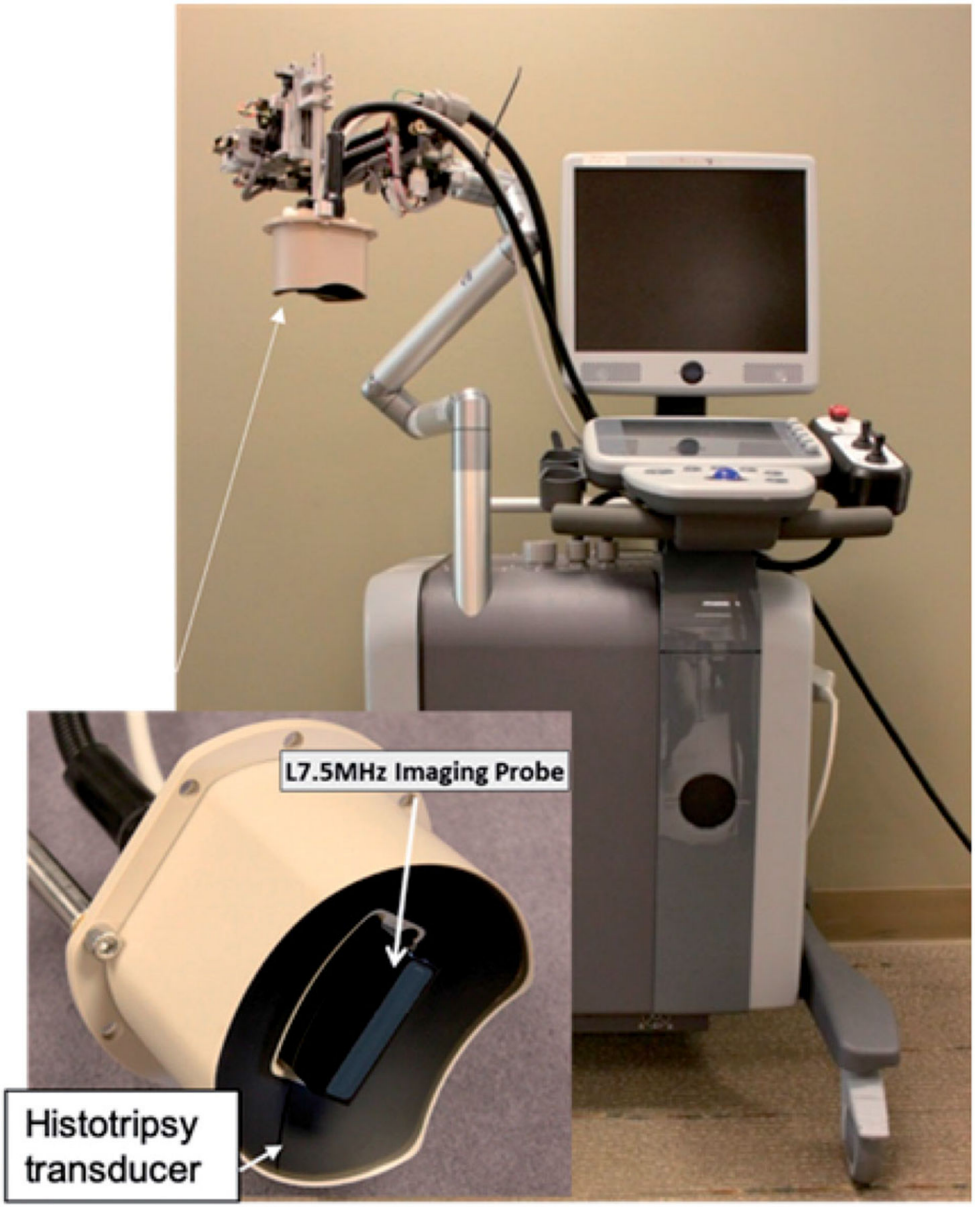
An ultrasound-guided histotripsy system that contains an ultrasound imaging engine, a histotripsy transducer with the imaging probe in the center (insert), a positioning arm that mechanically moves the histotripsy transducer.

In addition to an ultrasound transducer and electronic driver, a complete image-guided histotripsy system includes an ultrasound imaging engine, an ultrasound imaging probe, a motorized positioner or a robotic arm to precisely move the transducer and the imaging probe, and a coupling medium to ensure efficient ultrasound transmission from the transducer to the skin [17,98] (Figure 4). The ultrasound imaging probe is typically inserted in the center of the histotripsy transducer to image the plane containing the focal ablation zone. Histotripsy can also be guided by MRI [99], but this requires an MR-compatible ultrasound transducer and positioner.

To treat a target volume (e.g., a tumor), the target can be covered with overlapping focal zones by mechanically moving the ultrasound transducer with a motorized positioner or a robotic arm [37]. Alternatively, a phased array ultrasound transducer can be used to electronically steer the focus over the target volume by adjusting the phased or time delay of the signal input to each element [100]. A phased array transducer typically has hundreds to thousands of elements, and each element has independent driving electronics (essentially a separate ultrasound transducer), thus the phased array and associated driving electronics are much more complex compared to the single focus transducer.

## Image guidance

### Ultrasound

**Figure 5.**
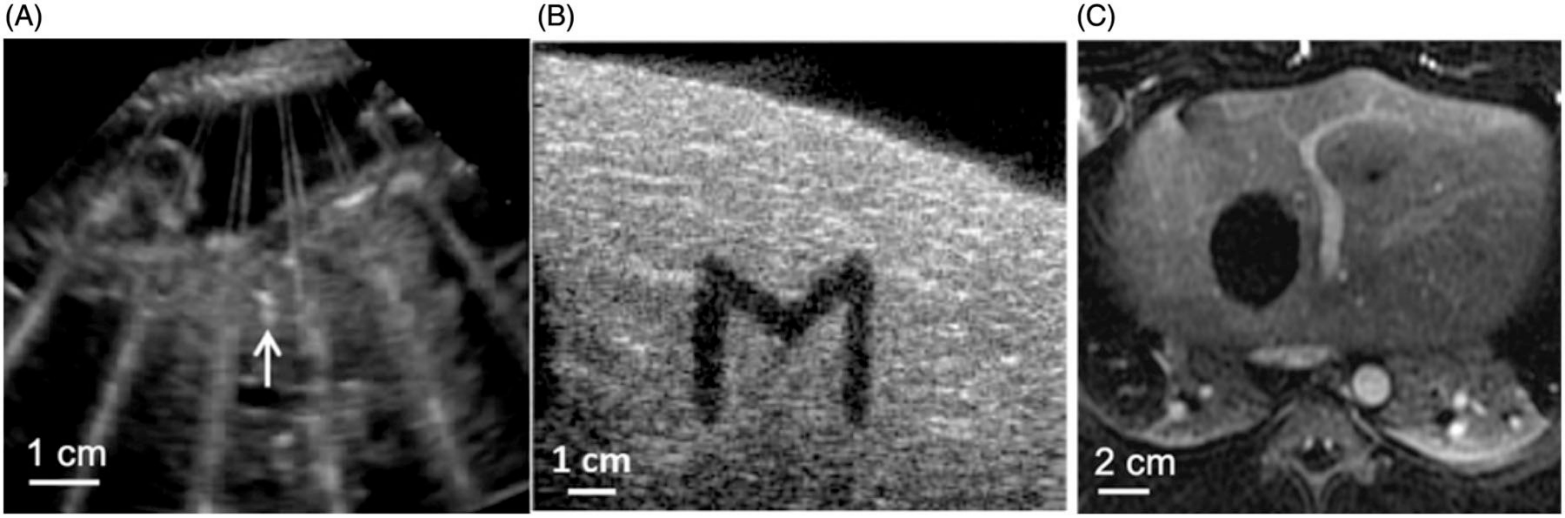
(A) Histotripsy-generated cavitation is seen as a hyperechoic zone on B-mode ultrasound. (B) ‘M’ shaped histotripsy lesion shows as a hypoechoic zone on B-mode ultrasound image. (C) Histotripsy ablation zone is seen as a hypointense (non-enhancing) region on this T1-weighted contrast-enhanced MR image.

Ultrasound imaging is typically used to guide histotripsy during treatment as cavitation can be visualized on B-mode ultrasound as a temporally changing (twinkling), hyperechoic (bright) zone (Figure 5(A)) [17,101]. For pretreatment targeting, the histotripsy focal position is marked on the ultrasound image and aligned with the target tissue by moving the transducer with a robotic arm or motorized positioner. The targeting depth can be varied by adjusting the depth of the transducer in the coupling medium between the transducer and skin. To confirm targeting accuracy, a short test pulse sequence is used to ensure that the hyperechoic cavitation zone is aligned with the target [84].

After targeting is confirmed, histotripsy treatment is delivered. The histotripsy focal position is moved mechanically or electronically to paint over the entire target volume. The overlying tissue and ultrasound attenuation vary across patients, even when the same ultrasound power is applied externally. Thus, the applied power is increased gradually until a cavitation bubble cloud is seen on ultrasound imaging, i.e., when the *in situ* focal pressure is just above the cavitation threshold [84,101]. This real-time feedback minimizes the energy deposition for histotripsy treatment and minimizes the risk of off-target cavitation or heating.

During treatment, the temporally changing hyperechoic cavitation zone grows larger with more flickering motion as the target volume tissue is gradually broken down into liquefied acellular debris. Post-treatment material appears as a hypoechoic (dark) zone on ultrasound imaging (Figure 5(B)) because the number and size of the sound scatterers decrease when the tissue is liquefied [102]. Histotripsy-induced tissue disruption can also be monitored by ultrasound elastography [98,103].

There are two main limitations of ultrasound imaging guidance. First, certain tumors can be seen clearly on MRI or CT, but not on ultrasound — often due to an insufficient diagnostic ultrasound window. In these cases, it is possible to co-register and/or fuze ultrasound images with pretreatment MRI or CT scans to increase the targeting accuracy. Second, 2 D ultrasound cannot provide 3 D volume imaging during treatments. However, 2 D ultrasound imaging is used for histotripsy guidance because the footprint of a 3 D ultrasound imaging probe is much larger, replacing some of the acoustic window space needed for the histotripsy therapy transducer.

### MRI

MRI can be used to evaluate histotripsy post-treatment effects [104]. Histotripsy ablation zones are clearly visualized on T1-weighted, T2-weighted, and contrast-enhanced MR images [47,84,85]. On T1-weighted images, ablation zones are hyperintense due to retained blood products. After contrast, ablation zones do not enhance, allowing differentiation of the ablation zone from residual tumor [105] (Figure 5(C)). For procedural imaging, histotripsy-induced cavitation is visible on MRI with special pulse-sequences synchronized to the ultrasound pulses, raising the possibility of future real-time monitoring [99].

## Pre-clinical applications

Histotripsy has been investigated for a wide range of preclinical applications in large and small animal models. The preclinical studies are summarized in the following sections based on the anatomic locations of the target tissue, which determines the transducer design and parameters used for histotripsy.

### Cancer (for tumors outside the brain)

This section summarizes the preclinical studies for tumors outside the brain, including a) liver cancer, b) prostate cancer, c) renal cancer, d) breast cancer e) pancreatic cancer, and f) musculoskeletal cancer. In addition, histotripsy has been shown to stimulate an immune response with a subsequent abscopal effect in animal tumor models, which is described in g) Immune Response. For the large animal (porcine, canine, and rabbit) models, frequencies between 700 kHz and 1 MHz were used. For small animal (rodent and murine) models, a 1 MHz small animal system was used. Other parameters are listed in Table 1.

### Liver cancer

**Figure 6.**
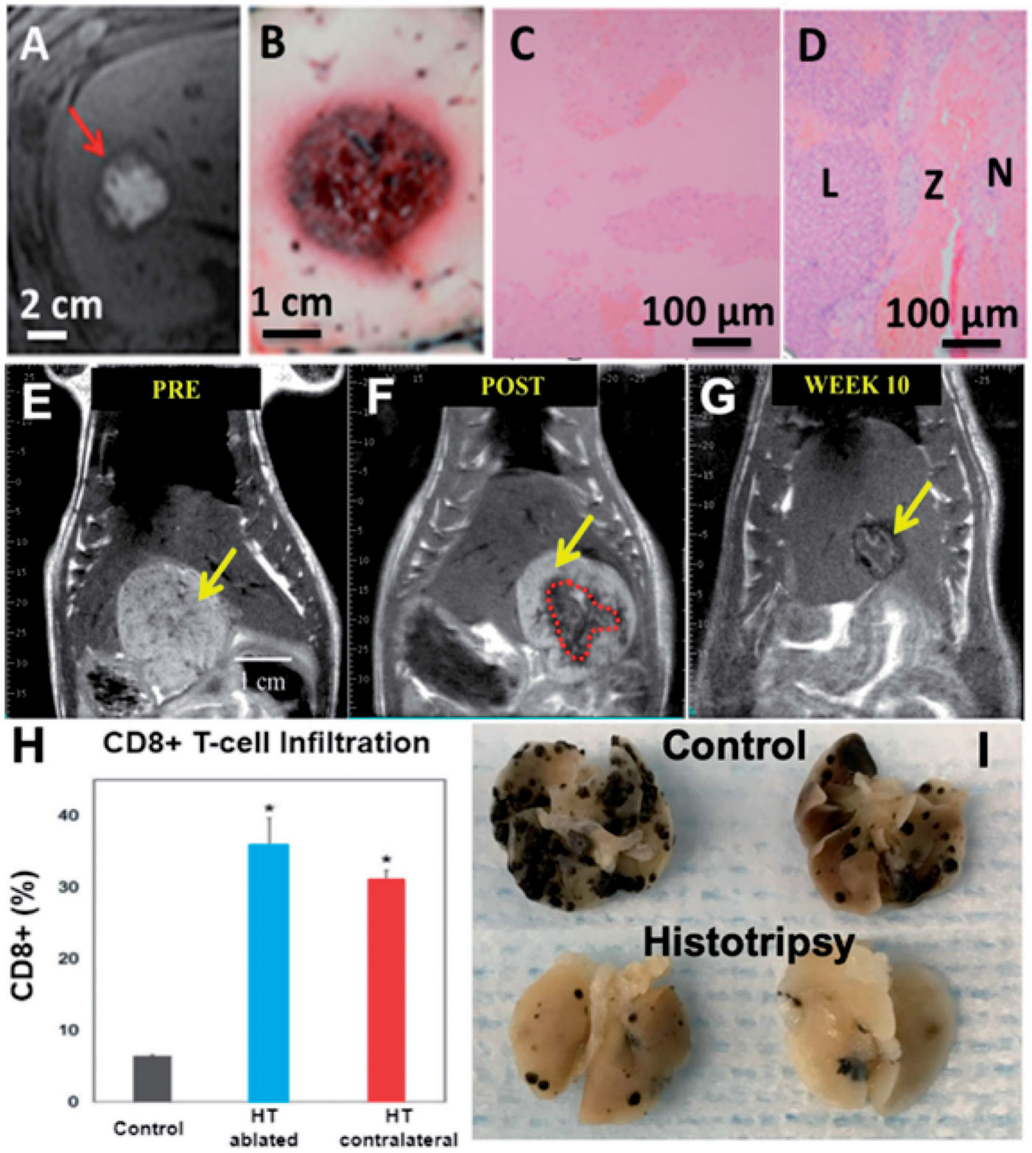
(A–D) Histotripsy treatment in the *in vivo* porcine liver. Axial T2-weighted MR images (A) and gross morphology (B) show the spherical ablation volume. (C) H&E slide of (100X) of the center of the ablation zone demonstrates no viable hepatocytes. (D) H&E slide (100X) of the periphery of the ablation shows viable liver (L), necrotic liver (N), and a thin zone of transition (Z). (E–G) MR images of partial histotripsy treatment in a rodent HCC model. (E) The appearance of the original untreated tumor on T2-weighted MRI was hyperintense (yellow arrow). (F) Post histotripsy, the ablation zone demonstrates T2 hypointense signal (red dashed lines). (G) At 12 weeks, only *a* ~5 mm non-tumoral fibrous tissue zone is detectable on MRI at the original tumor site, indicating near-complete resolution of the ablation zone and tumor. (H–I) Histotripsy induced immune response and abscopal effect (reproduction based on images from ref [39]. (H) FACS analysis of histotripsy-ablated (HT ablated) and contralateral non-ablated tumors (HT abscopal) identified comparable levels of intratumoral CD8^+^ T cell infiltration that is significantly higher than the untreated control tumors. (I) At gross examination, pulmonary metastases were reduced in mice treated with histotripsy compared to untreated controls.

Histotripsy has been shown to create effective and safe ablations in the *in vivo* human-scale porcine normal liver [37,38,84,85,101,106]. A target liver volume of 12–60 ml through 3–12 cm of overlying tissue was completely ablated in 20–75 min (Figure 6(A-D)). Within the ablated region, uniform tissue disruption was achieved with no intact cells remaining, while major vessels and bile ducts were intact.

As bones are highly reflective and absorptive for ultrasound propagation, the feasibility and safety of histotripsy through ribs was studied [84,101]. A focal pressure just above the cavitation threshold was used by gradually increasing the ultrasound power until cavitation was observed on ultrasound. The ablation zones created through full ribcage coverage vs. only overlying soft tissue had comparable dimensions. The highest temperature increase to ribs with the full ribcage covering the acoustic window was 4.1 ± 1.8 °C, which was not sufficient to cause thermal damage to the ribs or surrounding tissue. No body wall damage was observed on MRI.

In one paper by Smolock et al. [38], body wall damage was reported, likely due to pre-focal cavitation on the ribs, but this damage was eliminated in other studies [84,101] by lowering the focal pressure to just above the cavitation threshold and/or by lowering the duty cycle (to <1%). In certain cases, portal vein and hepatic vein thromboses were observed within the treatment zone [38], similar to radiofrequency and microwave ablation, although the thrombosis was observed to be transient with a complete resolution on follow-up imaging.

The longer-term response to the treatment of the liver by histotripsy was first studied in a normal rodent model [22]. The acellular homogenate generated by histotripsy was absorbed within 28 days, leaving only a sub-millimeter fibrous region at the treatment site. The biological response to tumor treatment has been studied in a rodent orthotopic, immune-competent liver tumor model (N1-S1) [41]. In 9/9 rats with complete tumor ablation and 5/6 rats with partial tumor ablation, the tumor was completely absorbed within 7–10 weeks post-histotripsy (Figure 6 (E-G)). Histology at 3 months showed a small zone of fibrosis at the ablation site with no evidence of residual tumor.

### Prostate cancer

Transabdominal histotripsy has been used to noninvasively liquefy prostate tissue in an *in vivo* canine normal prostate model [81,107-110] and an implanted canine prostate tumor model [44]. The number of histotripsy pulses or ultrasound pressure required to damage the urethra is much higher than prostate parenchyma or non-stromal prostate tumors [111]. As such, the target tumor or parenchyma tissue was completely liquefied to acellular debris while leaving the urethra and the prostate capsule intact. However, the urethra can also be purposely disintegrated when sufficiently high pressure and a high number of pulses were used, such that the liquefied acellular debris can be evacuated during urination. The enlarged flow channel was observed to re-endo-thelialize within 28 days [112].

As histotripsy causes mechanical tissue disruption, there is a concern that histotripsy may disrupt blood vessels and cause bleeding. Additionally, potential patient candidates for prostate ablation frequently are on long-term anticoagulant therapy. To investigate the risk of hemorrhage, histotripsy was used to treat the canine prostate in anticoagulated subjects. Results showed no adverse events and no clinically significant change in hemoglobin concentration post histotripsy [113].

### Renal cancer

Similar to the observations in the liver and prostate, histotripsy has been used to destroy renal tissue in large animal normal kidney models, while sparing the collecting system and urothelium immediately adjacent to or within the treatment zone [15,85,86]. Acellular debris was completely absorbed within 2 months, leaving only a small fibrous scar [23]. Histotripsy has also been used to effectively destroy tumors in a rabbit renal VX-2 tumor model [42].

### Breast cancer

Histotripsy has been shown to destroy breast tumors *in vitro* [74,114] and *in vivo* [115,116] in both subcutaneous and orthotopic mouse models. Results from histotripsy treatments in a 4T1 orthotopic mouse model showed histotripsy effectively destroyed the targeted regions of the breast tumors and led to innate immune cell infiltration, expression of pro-inflammatory cytokines, and a reduction in metastatic colonies in the 3 weeks following treatment, all correlating with an anti-tumor microenvironment [115,116]. Similar results have been observed for *in vitro* boiling histotripsy treatments of human breast adenocarcinoma cells which showed boiling histotripsy significantly increased secretions of damage-associated molecular patterns (CRT, HSP70, HMGB-1), pro-inflammatory cytokines (IFN-γ, IL-1α, IL-1 β, IL-18), and chemokines (IL-8) [114].

### Pancreatic cancer

Recent studies have explored histotripsy for the treatment of pancreatic cancer *in vivo* in both small [117,118] and large animal models [117]. Histotripsy was shown to effectively destroy Pan02 pancreatic tumors in an immunocompetent subcutaneous mouse model. This study further showed that histotripsy treatment of pancreatic tumors resulted in the release of tumor-associated proteins and DNA, which stimulated anti-tumor immune pathways and led to an inflammatory immune response (see Immune Response section below). Ongoing studies are investigating the potential of histotripsy for ablating pancreatic tumors in a novel SCID-like orthotopic pig tumor model that allows for human pancreatic tumors to be grown in the pancreas of immunocompromised pigs [117]. As part of this work [117], initial pilot studies in healthy pigs showed histotripsy could target and generate ablation within the pancreas of some subjects, with consistent targeting of the pancreas being difficult due to gaseous overlying tissues.

### Musculoskeletal tumors

Musculoskeletal tumors can originate from various soft tissues (muscle, cartilage) as well as bone. An *ex vivo* study of histotripsy using excised canine osteosarcoma tumors showed histotripsy could generate complete ablation of both the soft tissue components of the tumor as well as regions containing neoplastic cells surrounded by infiltrating osteoid matrix [119]. Using the same treatment parameters, histotripsy also showed no damage to *ex vivo* nerve and healthy bone samples, suggesting the potential of histotripsy for the tissue-selective ablation of musculoskeletal tumors in locations near critical structures [119]. Ongoing studies are investigating the *in vivo* feasibility of histotripsy for the treatment of spontaneous soft tissue sarcoma and osteosarcoma tumors in dogs, with initial results reporting that histotripsy can effectively ablate both types of musculoskeletal tumors.

### Immune response

Histotripsy has been shown to stimulate an innate and adaptive potent immune response in murine melanoma and liver tumor models [39]. This was manifested by an increase in CD8+ TIL cells, Neutrophil (Ly6G + cells), tumor-specific T cells, Dendritic cells (CD11c+), high-mobility group box 1 (HMGB1), and activated CD8+ T cells (as indicated by intracellular interferon gamma (IFNγ) expression)) at 7–10 days post-treatment (Figure 6(H)). The levels of the immune cell increase induced by histotripsy were significantly higher than seen for radiation therapy or radiofrequency ablation. Histotripsy also induced a strong systemic anti-tumor immune response and abscopal effect. Qu et al. [39] show that histotripsy of flank tumors significantly reduced pulmonary metastases compared to controls (*p* < .05) (Figure 6(I)). In addition, in a two-tumor model, histotripsy of one tumor inhibited the growth of contralateral untreated tumors. Boiling histotripsy has also been observed to stimulate an immune response in a renal tumor model [43]. These findings were hypothesized to be caused by the release of tumor-specific antigens from the histotripsy-produced acellular tumor debris and the immunological tumor death caused by histotripsy.

It is worth noting that prior studies show cavitation induced by lithotripsy can produce sparse to partial disruption of the target tumor in mouse tumor models. Some work indicated no negative impact on metastasis following lithotripsy tumor treatment [120-122], but an increased number of metastases was observed in other studies [123,124]. In these prior lithotripsy studies, various conditions of cavitation were created, resulting in different levels of tumor disruption, from sparse to complete which may lead to different systemic effects and impacts on distant tumors.

### Brain applications

For the noninvasive treatment of a target in the brain, ultrasound is delivered from outside the skull to focus inside the brain. Because the skull is highly absorptive and reflective of ultrasound, substantial attenuation and aberration occur for ultrasound propagation through the skull [125,126]. Typically, a hemispherical, large aperture, low frequency (~200kHz — 700 kHz), high-power ultrasound transducer is used for brain applications.

Transcranial MR-guided focused ultrasound (tcMRgFUS) thermal ablation uses continuous or long ultrasound exposures delivered from outside the skull to heat tissue inside the brain, guided by MR thermometry. TcMRgFUS thermal ablation has been FDA approved to treat essential tremor [127,128], and human trials are ongoing for treating centrally located brain tumors (NCT03028246). However, tcMRgFUS thermal ablation is limited to treating only central locations inside the brain and small targets (<1 cm) due to the potential for overheating of the skull. As histotripsy uses cavitation to achieve the tissue liquefaction and a low duty cycle (e.g., <0.1%) with intervening cooling time, limitations of treatment locations and volumes due to skull heating are less of a problem with transcranial histotripsy than with tcMRgFUS thermal ablation.

### Brain tumors

Histotripsy has been investigated as an option for noninvasive brain tumor treatment. First, the potential of using transcranial histotripsy to treat a wide range of locations and volumes through an excised human skull was studied. Hemispherical transcranial histotripsy transducer arrays (250 kHz and 500 kHz) with 256 elements and a focal distance of 15 cm were designed and constructed [129]. Using a 500 kHz array, 1-cycle pulses, and >30MPa peak negative pressure, 30 ml of *ex vivo* bovine brain was completely liquefied within 30 min through an excised human skull with electronic focal steering. This *ex vivo* model demonstrated that histotripsy was capable of ablating deep structures in the brain as well as at shallow locations (~5 mm from the skull surface). Thermocouple measurements in the skull showed that the temperature increase was maintained below 4 °C throughout a 60-min transcranial histotripsy treatment [130].

There is a concern that mechanical tissue disruption caused by histotripsy may induce hemorrhage or edema in the brain. This safety concern was investigated in the *in vivo* normal porcine brain [47]. As the pig skull is too flat and thick for ultrasound propagation, a portion of the pig skull was removed and histotripsy was delivered from outside the intact dura. Single focal and larger contiguous ablation zones were created in brain cortical tissue. MRI and histology at 2–4 h and 3 days after treatment showed that the target brain was liquefied to acellular debris. No hemorrhage or edema or other discernable damage was observed outside the treatment region, demonstrating initial safety for *in vivo* histotripsy brain treatment.

### Intracerebral hemorrhage (ICH)

ICH is a devastating form of hemorrhagic stroke caused by parenchymal clot formation due to the rupture of blood vessels in the brain [131]. Current treatments include open craniotomy [132,133] and a minimally invasive craniopuncture approach [134], where a catheter is inserted into the ICH through a small burr hole and thrombolytic drugs are injected. The lysed ICH is drained *via* the catheter over 3–7 days. The craniopuncture approach increases the risk of rebleeding and is often not effective for large hemorrhages [135,136]. Transcranial histotripsy was used to liquefy an *in vitro* clot quickly (within 30 min) through an excised human skull, and the liquefied clot was drained *via* a catheter without thrombolytic drugs. This approach has the potential to shorten the treatment time and the ICU stay for patients and potentially reduce ICH-related secondary brain injuries.

Gerhardson et al. showed that histotripsy can liquefy a large clot volume through an intact human skull as fast as 16 ml/min without overheating the skull [130]. Gerhardson et al. [137] also designed and built a catheter-based hydrophone, which allows for intra-procedural aberration correction. This catheter-based hydrophone combined with a neuronavigation system would allow the histotripsy ICH treatment to be performed without requiring an MRI scanner which makes it more suitable for an emergent procedure. The initial *in vivo* safety of histotripsy ICH treatment was demonstrated in an *in vivo* porcine ICH model [46]. Histotripsy was used to liquefy the ICH in the porcine brain, leaving a millimeter margin without damaging the surrounding brain tissue.

### Others

- ***Deep venous thrombosis (DVT)*** – DVT is the formation of clots in the deep veins in the body, often in the legs. Histotripsy was shown to noninvasively break down blood clots and recanalize blocked vessels using an external ultrasound transducer in *in vitro* blood clots [138] and an *in vivo* porcine DVT model [48,49,139]. The in vitro measurements showed that clot debris particles resulting from histotripsy were all smaller than 100 μm, with >99% smaller than 8 μm (size of a red blood cell). In the *in vivo* porcine DVT model, a flow channel confined within the vessel was created without damaging the vessel using intrinsic threshold histotripsy [49] or shock-scattering histotripsy at a relatively high frequency (2.5 MHz) [139]. Histotripsy was also effective in treating aged, retracted clots that were stiffer and less porous compared to acute clots, although requiring a higher number of ultrasound pulses [140].
- ***Neonatal hypoplastic heart syndrome –*** A congenital heart disease termed hyperplastic left heart syndrome requires the creation of a flow channel between the left and right chamber of the heart in neonates. Histotripsy was used to perforate the atrial septum in an open chest adult canine heart model [17] and the ventricular septum in neonatal swine through an intact chest [55,141].
- ***Calcified aortic stenosis –*** Calcified aortic stenosis is caused by calcific deposits on valve leaflets which cause a luminal narrowing and restricted blood flow to the body. Current treatments are either surgical aortic valve replacement (AVR) or transcatheter aortic valve replacement (TAVR). *In vivo* porcine experiments showed the safety and feasibility of using transthoracic histotripsy to noninvasively soften the aortic valve as a native valve repair without the need for a replacement valve [56,57].
- ***Fetal intervention –*** Histotripsy has been investigated to perform noninvasive fetal intervention for treatment and prevention of congenital heart diseases and fetal tumors in a pregnant sheep model [142,143].
- *Kidney stones –* Histotripsy has been shown to break down kidney stones into micron size debris [58,144]. In comparison, lithotripsy can fractionate kidney stones but often results in millimeter-sized fragments that can be difficult to spontaneously pass, and may result in urinary obstruction [145].
- ***Tendon*** – Although collagenous tissues have been shown to be more resistant to histotripsy-induced tissue damage, studies have shown that histotripsy can be used for the partial or complete ablation of tendons for the treatment of musculoskeletal conditions [56] and the noninvasive resection of the basal chordae in the treatment of ischemic mitral regurgitation [57].
- ***Bacteria and Biofilms –*** Biofilm formation on percutaneous and implanted biomedical devices is a common and significant clinical problem that leads to infection and other serious complications. Biomaterial-associated infections are a common complication following surgery resulting in serious and difficult-to-treat infections that often lead to graft removal. Histotripsy has been shown to destroy bacteria on biofilms used in hernia repair surgeries without damaging the surgical grafts [61,62,146-148]. Another common source of hospitalacquired infection is urinary catheters that often become contaminated with biofilms, resulting in catheter-associated urinary tract infections (CAUTIs) that adversely affect patient outcomes. A recent study demonstrated that histotripsy can precisely target luminal biofilms inside urinary catheters, resulting in complete removal of the biofilm and near-complete cell death of the associated bacteria cells (4-log reduction in colony-forming units per mL) [149]. Additional studies have also shown the potential for using histotripsy to kill bacteria in the treatment of abscesses in an *in vivo* porcine model, with results showing histotripsy liquefaction of the viscous pus and up to a 5-fold reduction in viable bacteria after treatment [150].

## Human trials

To date, three Phase I human trials have been conducted to investigate the safety of histotripsy in patients with benign prostatic hyperplasia, liver cancer, and calcific aortic stenosis.

### Benign prostatic hyperplasia (BPH)

In a Phase I safety trial at two medical centers in the US in 2016–2017 (NCT01896973) [64], a clinical prototype prostate histotripsy device (Vortx Rx) manufactured by HistoSonics (Ann Arbor, MI) was used to treat 25 patients with BPH, where an enlarged prostate constricts the urethra and causes difficulty with urination. No serious intraoperative adverse events were observed. Sixty-eight percent of the subjects underwent general anesthesia, whereas 32% were treated with sedation. Cavitation generation was observed by transrectal ultrasound imaging in all subjects, although tissue destruction was not observed. This is in contrast to the preclinical canine studies, where histotripsy liquefied the target prostate tissue, and the liquefied tissue was drained *via* a perforation created in the urethra by histotripsy resulting in effective debulking [81,108,109,111,113]. The International Prostate Symptom Score (IPSS) improved significantly compared to the pretreatment baseline, averaging 52.4% at 1 month, 50.8% at 3 months, and 44.0% at 6 months (*p* <.001). The outcomes were considered equivalent to results in patients treated with oral medication. Compared to the transabdominal ultrasound delivery in the canine prostate, ultrasound was delivered transperineally in humans, where there was more bone blockage and the prostate was located deeper to the skin surface. Thus, it is possible that the ultrasound pressure used in the human trial was not sufficient for effective tissue disruption due to the limited acoustic window. It is also possible that disruption of human prostate tissue requires a higher pressure or greater number of histotripsy pulses. This trial demonstrated initial safety in humans, yet the device and/or parameters need to be improved to demonstrate efficacy for prostatic histotripsy tissue disruption.

### Liver cancer

The first Phase I human trial of hepatic histotripsy in non-curative patients with multifocal liver malignancy was conducted in Barcelona, Spain in 2019 (NCT03741088). A clinical prototype hepatic histotripsy device manufactured by Histosonics, Inc. (Ann Arbor, MI) was used. Eleven tumors (range 0.5–2.1 cm) were targeted in 8 patients, including one hepatocellular carcinoma (HCC) and ten metastases (colorectal, breast, and gallbladder cancer) in the liver. No procedure-related significant adverse event occurred, demonstrating the early safety of hepatic histotripsy. All procedures met the primary endpoint, creation of an ablation per plan as assessed by MRI 1-day post-procedure. A single 5 mm tumor was mistargeted due to limitations in diagnostic ultrasound imaging, while in all other tumors local tumor regression was observed by MRI at 2 months. Volume contraction of the ablation zone averaged 36.0% at 1 week, 53.6% at 1 month, and 71.8% at 2 months, measured by T1-weighted contrasted MRI. Two patients (2/8, 25%), one each with HCC and colorectal cancer (CRC), had continuous post-procedure decline in tumor markers, and the patient with CRC had shrinkage of non-target tumors at 8 weeks post-procedure. This Phase I trial demonstrated the initial safety and efficacy of hepatic histotripsy and provides the first evidence of a potential abscopal effect induced by histotripsy in humans.

### Calcified aortic stenosis

Noninvasive ultrasound therapy of calcific aortic stenosis can be included in the histotripsy family at large as it relies on a similar range of pressures and pulses of tens of microseconds in duration. However, in contrast with conventional histotripsy which intends to liquefy soft tissue, noninvasive therapy of calcific valves is based on the softening of the calcified tissues to restore the mobility of the valve leaflet. The feasibility and safety of the approach were demonstrated in preclinical studies [56,57]. Next, a Phase I human trial of cardiac histotripsy was conducted in 10 patients with severe calcific aortic stenosis using Cardiawave’s Valvosoft device (NCT03779620) in France and the Netherlands in 2019 [65]. These patients were advanced in age (84.1 ± 6.5 years, 50% women), had severe comorbidities (8 with heart failure, 5 with coronary heart disease, and 5 with kidney failure), and were not eligible for percutaneous aortic valve replacement nor open-heart surgery. An external multi-element focused ultrasound transducer (bandwidth 700 kHz — 1.25 MHz) was used to deliver up to 60 min of histotripsy at a 100–300 Hz PRF, 0.25% duty cycle, and 15–20 MPa peak negative pressure to treat and soften the calcified regions of the aortic valve. At 1-month post-procedure, 6 patients had a mean increase of 27.6% in the aortic valve area (*p* = .03) and a mean pressure gradient decrease of 23.5% (*p* = 0.03), and no patients experienced significant adverse effects including cerebrovascular accidents, deterioration of cognitive function, and no deaths occurred. In total, 6 patients were classified as responders and 4 as non-responders. For the 6 responder patients, the applied ultrasound energy and the treatment duration were greater compared to the other 4 patients. At 6 months, the treatment effect for the aortic valve area was maintained in the responder group. At 1-year follow-up, four patients had died due to progression of heart failure not linked to the histotripsy procedure and one had undergone a percutaneous aortic valve replacement, while the remaining five patients had no major adverse effects related to the procedure. This trial suggests that noninvasive therapy of calcific aortic stenosis is safe and feasible.

## Advantages and limitations

Histotripsy precisely controls acoustic cavitation to mechanically destroy target tissue into acellular debris that is absorbed by the body. There are substantial differences between histotripsy and the clinical standard thermal ablation methods such as radiofrequency ablation and microwave ablation, resulting in both unique advantages and limitations as summarized below.

### Advantages

- **Noninvasiveness –** Histotripsy uses externally delivered transcutaneous ultrasound and does not require incisions or punctures. This characteristic reduces risks commonly associated with percutaneous devices such as hemorrhage, organ damage, and tumor seeding along the needle insertion track.
- **Tissue removal/debulking** – Histotripsy mechanically liquefies the target tissue into acellular debris, which is absorbed by the body and results in effective tissue removal. This enables histotripsy to be used in applications where tissue removal is required, such as thrombosis. Preclinical animal studies and a pilot human trial showed that the treated volume of tissue is absorbed within 1–2 months [22,23]. This rapid involution of the ablation zone may simplify the detection of incompletely treated or recurrent tumors.
- **Tissue selectivity** – The ultrasound pressure and the number of histotripsy pulses to destroy collagen-based tissue or tissue with greater mechanical strength (such as bile ducts, normal large vessels, bowel, nerves, collecting system, urethra) is much higher than non-collagenous tissues (such as parenchymal organs and tumors) [69]. With this differential threshold, appropriate ultrasound parameters can be chosen to safely apply histotripsy at locations near critical structures to ablate the target tumor while preserving adjacent critical structures.
- **Sharp boundary** – Histotripsy produces a binary effect in which cavitation-induced damage only occurs when the ultrasound pressure exceeds the cavitation threshold [68]. Therefore, the transition zone of partial damage between the acellular debris inside the histotripsy ablation zone and surrounding viable cells is very narrow (a few hundred microns in non-mobile tissues). The boundary of a thermal ablation zone includes a transition zone of partially damaged tissue between the necrotic and viable tissue, which is often several millimeters or wider due to a heat gradient. This zone of partial ablation can lead to incomplete tumor kill.
- **Avoidance of hemorrhage** – Published large animal studies have demonstrated no post-histotripsy bleeding risk in vascular organs, even in anticoagulated subjects [113] or when the target region contains major vessels [37,106]. The etiology of histotripsy-induced coagulation is believed to be two-fold. First, large vessels contain significant amounts of collagen making them more resistant to histotripsy-induced damage. Second, cavitation generated during histotripsy is known to promote coagulation by inducing platelet activation and aggregation.
- **Imaging feedback** – Histotripsy is guided and monitored by real-time ultrasound imaging. This real-time imaging feedback allows treatment monitoring, adjustment of treatment parameters for an individual patient, and evaluation of the treatment effect immediately post-treatment. This unique feature ensures high treatment accuracy and efficacy.
- **Immune response** – Histotripsy has been shown to stimulate a potent immune and abscopal response in preclinical animal tumor models [39] and potentially in the Phase I liver cancer human trial. In animals, this immune response led to the regression of the residual untreated tumor, reduction of metastases, and enhancement of immunotherapy. Even though the mechanism is not clearly understood, the global treatment effect beyond the local tumor ablation may bring additional benefits and improve clinical outcomes for cancer patients.

### Limitations

- **Organs containing gas** – Some organs, such as the lung and the gastrointestinal tract, contain gas and may not be suitable targets for histotripsy. Because the cavitation threshold is very low in gas-containing organs, histotripsy could result in extensive collateral damage to surrounding normal tissue.
- **Acoustic access** – Histotripsy requires very high ultrasound pressures, and the achievable pressure is proportional to the transducer aperture size not blocked by bones (e.g., ribs) or gas (e.g., lung). Therefore, some locations in the body cannot be treated by histotripsy with externally delivered ultrasound, such as the central lung and potentially the pancreas, due to gas or bone blockage. There is also a limitation on the depth of the tissue that can be treated by externally delivered histotripsy due to the increasing ultrasound attenuation with increasing tissue depth which may preclude use on very obese subjects.
- **Thrombosis** – Histotripsy may induce thrombosis in vessels within the treated region, likely due to platelet activation and aggregation from cavitation [84]. This thrombosis effect is also observed to some degree following radiofrequency ablation and microwave ablation [85,151,152].
- **Risk of metastases** – As histotripsy mechanically disrupts target tissue, there is a theoretical risk that histotripsy may dislodge and release tumor cells from the target tumor, thus leading to an increased risk of metastases. However, studies to date have instead shown no increase [42] or a reduced risk of metastases following histotripsy, likely due to a concomitant immune response [39].

## Summary

Histotripsy is a noninvasive, non-ionizing, and non-thermal ablation technology that is based on ultrasound and guided by real-time imaging. With acoustic cavitation as the primary mechanism, histotripsy displays unique features that distinguish it from current ablation technologies, including physical tissue removal, tissue-selective ablation, and a precise boundary. Histotripsy can be performed with real-time imaging feedback and early animal studies demonstrate stimulation of the immune system. Phase I human trials have shown the initial safety and efficacy of histotripsy to treat patients with malignant liver tumors, BPH, and calcified aortic stenosis. Despite substantial technical, preclinical, and clinical progress to date, there is a large amount of future work necessary for technical development, preclinical research, and human studies before histotripsy can become a widespread clinical treatment modality.

## Figures and Tables

**Table 1.** Typical histotripsy parameters.

Parameters	Intrinsic Threshold Histotripsy	Shock-scattering Histotripsy	Boiling Histotripsy	HIFU
*Frequency*	250kHz–3MHz	500kHz–3MHz	1–3 MHz	1–5 MHz
*Pulse duration*	1–2 cycles 0.5–4μs	3–10 cycles	100–200 1–20 ms	Continuous waves or high duty cycle
*P*−	>26 MPa	15–25 MPa	10–20 MPa	5–10 MPa
*P*+	No requirement	>50Mpa	>70MPa	5–30 MPa
*Duty cycle* *	≤1%	≤1%	≤2%	10–100%
*PRF*	1Hz – 1kHz	1Hz–1kHz	1Hz–2Hz	–
*I_SPPA_* **	>30 kW/cm^2^	9–40 kW/cm^2^	8–30 kW/cm^2^	0.5–10 kW/cm^2^
*I_SPTA_* ***	0.5–300 W/cm^2^	1–400 W/cm^2^	50–600 W/cm^2^	100–5000 W/cm^2^
*Number of pulses*	50–2000	50–2000	1–100	–
*Bioeffect*	Mechanical tissue liquefaction		Mechanical tissue liquefaction	Thermal necrosis
*Mechanism*	Inertial cavitation		Boiling cavitation	Thermal

* Duty cycle: ultrasound on-time/total treatment time

** I_SPPA_: Spatial peak pulse average intensity

*** I_SPTA_: Spatial peak time average intensity.

## References

[1] Fakiris AJ, McGarry RC, Yiannoutsos CT, Stereotactic body radiation therapy for early-stage non-small-cell lung carcinoma: four-year results of a prospective phase II study. Int J Radiat Oncol Biol Phys. 2009;75(3):677–682. 19251380 10.1016/j.ijrobp.2008.11.042

[2] Lo SS, Fakiris AJ, Chang EL, Stereotactic body radiation therapy: a novel treatment modality. Nat Rev Clin Oncol. 2010;7(1):44–54. 19997074 10.1038/nrclinonc.2009.188

[3] Kudo M. Radiofrequency ablation for hepatocellular carcinoma: updated review in 2010. Oncology. 2010;78(1):113–124. 20616593 10.1159/000315239

[4] Martin RC, Scoggins CR, McMasters KM. Safety and efficacy of microwave ablation of hepatic tumors: a prospective review of a 5-year experience. Ann Surg Oncol. 2010;17(1):171–178. 19707829 10.1245/s10434-009-0686-z

[5] Lanza E, Palussiere J, Buy X, Percutaneous image-guided cryoablation of breast cancer: a systematic review. J Vasc Interv Radiol. 2015;26(11):1652–1657. 26342882 10.1016/j.jvir.2015.07.020

[6] Marrero JA, Pelletier S. Hepatocellular carcinoma. Clin Liver Dis. 2006;10(2):339–351. 16971265 10.1016/j.cld.2006.05.012

[7] Giorgio A, Tarantino L, de Stefano G, Complications after percutaneous saline-enhanced radiofrequency ablation of liver tumors: 3-year experience with 336 patients at a single center. AJR Am J Roentgenol. 2005;184(1):207–211. 15615976 10.2214/ajr.184.1.01840207

[8] Livraghi T, Solbiati L, Meloni MF, Treatment of focal liver tumors with percutaneous radio-frequency ablation: complications encountered in a multicenter study. Radiology. 2003;226(2):441–451. 12563138 10.1148/radiol.2262012198

[9] Khokhlova TD, Hwang JH. HIFU for palliative treatment of pancreatic cancer. Adv Exp Med Biol. 2016;880:83–95. 26486333 10.1007/978-3-319-22536-4_5

[10] Haar GT, Coussios C. High intensity focused ultrasound: physical principles and devices. Int J Hyperthermia. 2007;23(2):89–104. 17578335 10.1080/02656730601186138

[11] Zibari GB, Riche A, Zizzi HC, Surgical and nonsurgical management of primary and metastatic liver tumors. Am Surg. 1998;64(3):211–220. 9520809

[12] McGhana JP, Dodd GD 3rd. Radiofrequency ablation of the liver: current status. AJR Am J Roentgenol. 2001;176(1):3–16. 11133529 10.2214/ajr.176.1.1760003

[13] Fukuda H, Ito R, Ohto M, Treatment of small hepatocellular carcinomas with US-guided high-intensity focused ultrasound. Ultrasound Med Biol. 2011;37(8):1222–1229. 21645963 10.1016/j.ultrasmedbio.2011.04.020

[14] Xu Z, Ludomirsky A, Eun LY, Controlled ultrasound tissue erosion. IEEE Trans Ultrason Ferroelectr Freq Control. 2004;51(6):726–736. 15244286 10.1109/tuffc.2004.1308731 PMC2669757

[15] Roberts WW, Hall TL, Ives K, Pulsed cavitational ultrasound: a noninvasive technology for controlled tissue ablation (histotripsy) in the rabbit kidney. J Urol. 2006;175(2):734–738. 16407041 10.1016/S0022-5347(05)00141-2

[16] Parsons JE, Cain CA, Abrams GD, Pulsed cavitational ultrasound therapy for controlled tissue homogenization. Ultrasound Med Biol. 2006;32(1):115–129. 16364803 10.1016/j.ultrasmedbio.2005.09.005

[17] Xu Z, Owens G, Gordon D, Noninvasive creation of an atrial septal defect by histotripsy in a canine model. Circulation. 2010;121(6):742–749. 20124126 10.1161/CIRCULATIONAHA.109.889071 PMC2834201

[18] ter Haar GR. High intensity focused ultrasound for the treatment of tumors. Echocardiography. 2001;18(4):317–322. 11415504 10.1046/j.1540-8175.2001.00317.x

[19] Khokhlova VA, Fowlkes JB, Roberts WW, Histotripsy methods in mechanical disintegration of tissue: towards clinical applications. Int J Hyperthermia. 2015;31(2):145–162. 25707817 10.3109/02656736.2015.1007538 PMC4448968

[20] Crum LA, Fowlkes JB. Acoustic cavitation generated by microsecond pulses of ultrasound. Nature. 1986;319(6048):52–54.

[21] Movahed P, Kreider W, Maxwell AD, Cavitation-induced damage of soft materials by focused ultrasound bursts: a fracture-based bubble dynamics model. J Acoust Soc Am. 2016;140(2):1374. 27586763 10.1121/1.4961364 PMC5848835

[22] Vlaisavljevich E, Greve J, Cheng X, Non-invasive ultrasound liver ablation using histotripsy: chronic study in an *in vivo* rodent model. Ultrasound Med Biol. 2016;42(8):1890–1902. 27140521 10.1016/j.ultrasmedbio.2016.03.018 PMC4912895

[23] Hall TL, Kieran K, Ives K, Histotripsy of rabbit renal tissue *in vivo*: temporal histologic trends. J Endourol. 2007;21(10):1159–1166. 17949317 10.1089/end.2007.9915

[24] Fry FJ, Kossoff G, Eggleton RC, Threshold ultrasonic dosages for structural changes in the mammalian brain. J Acoust Soc Am. 1970;48 (6):Suppl 2:1413+. 10.1121/1.1912301 5489906

[25] Senapati N, Lele PP, Caulfield JB. On mechanisms of cavitational damage to biological tissues. J. Acoust. Soc. Am 1974;55:1919507.

[26] Newman R, Hackett R, Senior D, Pathologic effects of ESWL on canine renal tissue. Urology. 1987;29(2):194–200. 3811098 10.1016/0090-4295(87)90152-x

[27] Delius M, Enders G, Xuan ZR, Biological effects of shock waves: kidney damage by shock waves in dogs–dose dependence. Ultrasound Med Biol. 1988;14(2):117–122. 3347964 10.1016/0301-5629(88)90178-0

[28] Weiss N, Delius M, Gambihler S, Influence of the shock wave application mode on the growth of A-Mel 3 and SSK2 tumors in vivo. Ultrasound Med Biol. 1990;16(6):595–605. 2238268 10.1016/0301-5629(90)90025-8

[29] Delius M, Denk R, Berding C, Biological effects of shock waves: cavitation by shock waves in piglet liver. Ultrasound Med Biol. 1990;16(5):467–472. 2238253 10.1016/0301-5629(90)90169-d

[30] Gambihler S, Delius M, Brendel W. Biological effects of shock waves: cell disruption, viability, and proliferation of L1210 cells exposed to shock waves *in vitro*. Ultrasound Med Biol. 1990;16(6):587–594. 2238267 10.1016/0301-5629(90)90024-7

[31] Tavakkoli J, Birer A, Arefiev A, A piezocomposite shock wave generator with electronic focusing capability: application for producing cavitation-induced lesions in rabbit liver. Ultrasound Med Biol. 1997;23(1):107–115. 9080623 10.1016/s0301-5629(96)00175-5

[32] Arefiev A, Prat F, Chapelon JY, Ultrasound-induced tissue ablation: studies on isolated, perfused porcine liver. Ultrasound Med Biol. 1998;24(7):1033–1043. 9809637 10.1016/s0301-5629(98)00046-5

[33] Vasquez JM, Eisenberg E, Osteen KG, Laparoscopic ablation of endometriosis using the cavitational ultrasonic surgical aspirator. J Am Assoc Gynecol Laparosc. 1993;1(1):36–42. 9050458 10.1016/s1074-3804(05)80756-x

[34] Prat F, Chapelon JY, Abou el Fadil F, Focused liver ablation by cavitation in the rabbit: a potential new method of extracorporeal treatment. Gut. 1994;35(3):395–400. 8150355 10.1136/gut.35.3.395 PMC1374598

[35] Rosenschein U, Yakubov SJ, Guberinich D, Shock-wave thrombus ablation, a new method for noninvasive mechanical thrombolysis. Am J Cardiol. 1992;70(15):1358–1361. 1442591 10.1016/0002-9149(92)90775-t

[36] Topaz M, Motiei M, Assia E, Acoustic cavitation in phacoe-mulsification: chemical effects, modes of action and cavitation index. Ultrasound Med Biol. 2002;28(6):775–784. 12113790 10.1016/s0301-5629(02)00514-8

[37] Vlaisavljevich E, Kim Y, Allen S, Image-guided non-invasive ultrasound liver ablation using histotripsy: feasibility study in an *in vivo* porcine model. Ultrasound Med Biol. 2013;39(8):1398–1409. 23683406 10.1016/j.ultrasmedbio.2013.02.005 PMC3709011

[38] Smolock AR, Cristescu MM, Vlaisavljevich E, Robotically assisted sonic therapy as a noninvasive nonthermal ablation modality: proof of concept in a porcine liver model. Radiology. 2018;287(2):485–493. 29381870 10.1148/radiol.2018171544

[39] Qu S, Worlikar T, Felsted AE, Non-thermal histotripsy tumor ablation promotes abscopal immune responses that enhance cancer immunotherapy. J Immunother Cancer. 2020;8(1):e000200. 31940590 10.1136/jitc-2019-000200 PMC7057529

[40] Khokhlova TD, Schade GR, Wang Y-N, Pilot *in vivo* studies on transcutaneous boiling histotripsy in porcine liver and kidney. Sci Rep. 2019;9(1):20176. 31882870 10.1038/s41598-019-56658-7 PMC6934604

[41] Worlikar T, Mendiratta-Lala M, Vlaisavljevich E, Effects of histotripsy on local tumor progression in an *in vivo* orthotopic rodent liver tumor model. BME Front. 2020;2020:1–14. 10.34133/2020/9830304 PMC8318009 34327513

[42] Styn NR, Hall TL, Fowlkes JB, Histotripsy of renal implanted VX-2 tumor in a rabbit model: investigation of metastases. Urology. 2012;80(3):724–729. 22925247 10.1016/j.urology.2012.06.020

[43] Schade GR, Wang Y-N, D’Andrea S, Boiling histotripsy ablation of renal cell carcinoma in the Eker rat promotes a systemic inflammatory response. Ultrasound Med Biol. 2019;45(1):137–147. 30340920 10.1016/j.ultrasmedbio.2018.09.006 PMC6546431

[44] Schade GR, Keller J, Ives K, Histotripsy focal ablation of implanted prostate tumor in an ACE-1 canine cancer model. J Urol. 2012;188(5):1957–1964. 22999534 10.1016/j.juro.2012.07.006 PMC4296596

[45] Hempel CR, Hall TL, Cain CA, Histotripsy fractionation of prostate tissue: local effects and systemic response in a canine model. J Urol. 2011;185(4):1484–1489. 21334667 10.1016/j.juro.2010.11.044 PMC3075837

[46] Gerhardson T, Sukovich JR, Chaudhary N, Histotripsy clot liquefaction in a porcine intracerebral hemorrhage model. Neurosurgery. 2020;86(3):429–436. 30924501 10.1093/neuros/nyz089 PMC7308653

[47] Sukovich JR, Cain CA, Pandey AS, *In vivo* histotripsy brain treatment. J Neurosurg. 2018;2018:1–8. 10.3171/2018.4.JNS172652 PMC6925659 30485186

[48] Maxwell AD, Owens G, Gurm HS, Noninvasive treatment of deep venous thrombosis using pulsed ultrasound cavitation therapy (histotripsy) in a porcine model. J Vasc Interv Radiol. 2011;22(3):369–377. 21194969 10.1016/j.jvir.2010.10.007 PMC3053086

[49] Zhang X, Macoskey JJ, Ives K, Non-invasive thrombolysis using microtripsy in a porcine deep vein thrombosis model. Ultrasound Med Biol. 2017;43(7):1378–1390. 28457630 10.1016/j.ultrasmedbio.2017.01.028 PMC5440202

[50] Bollen V, Hendley SA, Paul JD, *In vitro* thrombolytic efficacy of single- and five-cycle histotripsy pulses and rt-PA. Ultrasound Med Biol. 2020;46(2):336–349. 31785841 10.1016/j.ultrasmedbio.2019.10.009 PMC6930350

[51] Bader K, Hendley SA, Bollen V. Assessment of Collaborative Robot (Cobot)-assisted histotripsy for venous clot ablation. IEEE Trans Biomed Eng. 2020.;68(4):1220–1228 10.1109/TBME.2020.3023630 PMC8018710 32915723

[52] Khokhlova TD, Monsky WL, Haider YA, Histotripsy liquefaction of large hematomas. Ultrasound Med Biol. 2016;42(7):1491–1498. 27126244 10.1016/j.ultrasmedbio.2016.01.020 PMC4899253

[53] Khokhlova TD, Kucewicz JC, Ponomarchuk EM, Effect of stiffness of large extravascular hematomas on their susceptibility to boiling histotripsy liquefaction *in vitro*. Ultrasound Med Biol. 2020;46(8):2007–2016. 32444137 10.1016/j.ultrasmedbio.2020.04.023 PMC7360281

[54] Li Y, Liu Y, Li R, Histotripsy liquefaction of large hematoma for intracerebral hemorrhage using millisecond-length ultrasound pulse groups combined with fundamental and second harmonic superposition: a preliminary study. Ultrasound Med Biol. 2020;46(5):1244–1257. 32111458 10.1016/j.ultrasmedbio.2020.01.026

[55] Owens GE, Miller RM, Owens ST, Intermediate-term effects of intracardiac communications created noninvasively by therapeutic ultrasound (histotripsy) in a porcine model. Pediatr Cardiol. 2012;33(1):83–89. 21910018 10.1007/s00246-011-0094-6

[56] Messas E, Rémond MC, Goudot G, Feasibility and safety of non-invasive ultrasound therapy (NIUT) on an porcine aortic valve. Phys Med Biol. 2020;65(21):215004. 33104523 10.1088/1361-6560/aba6d3

[57] Villemain O, Robin J, Bel A, Pulsed cavitational ultrasound softening: a new non-invasive therapeutic approach of calcified bioprosthetic valve stenosis. JACC Basic Transl Sci. 2017;2(4):372–383. 29367953 10.1016/j.jacbts.2017.03.012 PMC5777603

[58] Duryea AP, Hall TL, Maxwell AD, Histotripsy erosion of model urinary calculi. J Endourol/Endour Soc. 2011;25(2):341–344. 10.1089/end.2010.0407 PMC3701315 21091223

[59] Matula TJ, Wang Y-N, Khokhlova T, Treating porcine abscesses with histotripsy: a pilot study. Ultrasound Med Biol. 2021;47(3):603–619. 33250219 10.1016/j.ultrasmedbio.2020.10.011 PMC7855811

[60] Smallcomb M, Simon JC. Investigation into tendon histotripsy. J Acoust Soc Am. 2019;145(3):1862–1862.

[61] Bigelow TA, Northagen T, Hill TM, The destruction of *Escherichia coli* biofilms using high-intensity focused ultrasound. Ultrasound Med Biol. 2009;35(6):1026–1031. 19171416 10.1016/j.ultrasmedbio.2008.12.001

[62] Bigelow TA, Thomas CL, Wu H, Histotripsy treatment of *S. Aureus* biofilms on surgical mesh samples under varying pulse durations. IEEE Trans Ultrason Ferroelectr Freq Control. 2017;64(10):1420–1428. 28650808 10.1109/TUFFC.2017.2718841 PMC5819746

[63] Bigelow TA, Thomas CL, Wu H, Impact of high-intensity ultrasound on strength of surgical mesh when treating biofilm infections. IEEE Trans Ultrason Ferroelectr Freq Con. 2019;66(1):38–44. 10.1109/TUFFC.2018.2881358 PMC6378954 30442604

[64] Schuster TG, Wei JT, Hendlin K, Histotripsy treatment of benign prostatic enlargement using the Vortx Rx system: initial human safety and efficacy outcomes. Urology. 2018;114:184–187. 29330000 10.1016/j.urology.2017.12.033

[65] Messas E, IJsselmuiden JA, Goudot G Feasibility and performance of noninvasive ultrasound therapy in patients with severe symptomatic aortic valve stenosis: a first-in-human study. Circulation 2021;143:968–970. 33486971 10.1161/CIRCULATIONAHA.120.050672

[66] Pahk KJ, Gelat P, Kim H, Bubble dynamics in boiling histotripsy. Ultrasound Med Biol. 2018;44(12):2673–2696. 30228043 10.1016/j.ultrasmedbio.2018.07.025

[67] Bader KB, Vlaisavljevich E, Maxwell AD. For whom the bubble grows: physical principles of bubble nucleation and dynamics in histotripsy ultrasound therapy. Ultrasound Med Biol. 2019;45(5):1056–1080. 30922619 10.1016/j.ultrasmedbio.2018.10.035 PMC6524960

[68] Maxwell AD, Cain CA, Hall TL, Probability of cavitation for single ultrasound pulses applied to tissues and tissue-mimicking materials. Ultrasound Med Biol. 2013;39(3):449–465. 23380152 10.1016/j.ultrasmedbio.2012.09.004 PMC3570716

[69] Mancia L, Vlaisavljevich E, Yousefi N, Modeling tissue-selective cavitation damage. Phys Med Biol. 2019;64(22):225001 31639778 10.1088/1361-6560/ab5010 PMC6925591

[70] Mancia L, Vlaisavljevich E, Xu Z, Predicting tissue susceptibility to mechanical cavitation damage in therapeutic ultrasound. Ultrasound Med Biol. 2017;43(7):1421–1440. 28408061 10.1016/j.ultrasmedbio.2017.02.020

[71] Vlaisavljevich E, Lin K-W, Maxwell A, Effects of ultrasound frequency and tissue stiffness on the histotripsy intrinsic threshold for cavitation. Ultrasound Med. Biol. 2015;41(6):1651–1667. 25766571 10.1016/j.ultrasmedbio.2015.01.028 PMC4426049

[72] Maxwell AD, Wang T-Y, Cain CA, Cavitation clouds created by shock scattering from bubbles during histotripsy. J Acoust Soc Am. 2011;130(4):1888–1898. 21973343 10.1121/1.3625239 PMC3206907

[73] Vlaisavljevich E, Maxwell A, Mancia L, Visualizing the histotripsy process: bubble cloud-cancer cell interactions in a tissue-mimicking environment. Ultrasound Med Biol. 2016;42(10):2466–2477. 27401956 10.1016/j.ultrasmedbio.2016.05.018 PMC5010997

[74] Wang YN, Khokhlova T, Bailey M, Histological and biochemical analysis of mechanical and thermal bioeffects in boiling histotripsy lesions induced by high intensity focused ultrasound. Ultrasound Med Biol. 2013;39(3):424–438. 23312958 10.1016/j.ultrasmedbio.2012.10.012 PMC3570648

[75] Winterroth F, Xu Z, Wang T-Y, Examining and analyzing subcellular morphology of renal tissue treated by histotripsy. Ultrasound Med Biol. 2011;37(1):78–86. 21144960 10.1016/j.ultrasmedbio.2010.10.002 PMC3038584

[76] Zhou Y, Wang X. Effect of pulse duration and pulse repetition frequency of cavitation histotripsy on erosion at the surface of soft material. Ultrasonics. 2018;84:296–309. 29182946 10.1016/j.ultras.2017.11.012

[77] Xu Z, Fan Z, Hall TL, Size measurement of tissue debris particles generated from pulsed ultrasound cavitational therapy-histotripsy. Ultrasound Med. Biol 2009;35(2):245–255. 19027218 10.1016/j.ultrasmedbio.2008.09.002 PMC2706707

[78] Vlaisavljevich E, Kim Y, Owens G, Effects of tissue mechanical properties on susceptibility to histotripsy-induced tissue damage. Phys Med Biol. 2014;59(2):253–270. 24351722 10.1088/0031-9155/59/2/253 PMC4888779

[79] Macoskey JJ, Zhang X, Hall TL, Bubble-induced color doppler feedback correlates with histotripsy-induced destruction of structural components in liver tissue. Ultrasound Med Biol. 2018;44(3):602–612. 29329687 10.1016/j.ultrasmedbio.2017.11.012 PMC5801099

[80] Mancia L, Rodriguez M, Sukovich J, Single-bubble dynamics in histotripsy and high-amplitude ultrasound: modeling and validation. Phys Med Biol. 2020;65(22):225014. 33179611 10.1088/1361-6560/abb02b

[81] Styn N, Hall TL, Fowlkes JB, Histotripsy homogenization of the prostate: thresholds for cavitation damage of periprostatic structures. J Endourol. 2011;25(9):1531–1535. 21815807 10.1089/end.2010.0648 PMC3168978

[82] Vlaisavljevich E, Lin K-W, Warnez MT, Effects of tissue stiffness, ultrasound frequency, and pressure on histotripsy-induced cavitation bubble behavior. Phys Med Biol. 2015;60(6):2271–2292. 25715732 10.1088/0031-9155/60/6/2271 PMC4360891

[83] Vlaisavljevich E, Maxwell A, Warnez M, Histotripsy-induced cavitation cloud initiation thresholds in tissues of different mechanical properties. IEEE Trans Ultrason Ferroelectr Freq Control. 2014;61(2):341–352. 24474139 10.1109/TUFFC.2014.6722618 PMC4158820

[84] Knott EA, Swietlik JF, Longo KC, Robotically-assisted sonic therapy for renal ablation in a live porcine model: initial preclinical results. J Vasc Interv Radiol. 2019;30(8):1293–1302. 31130365 10.1016/j.jvir.2019.01.023 PMC6925588

[85] Longo KC, Knott EA, Watson RF, Robotically Assisted Sonic Therapy (RAST) for noninvasive hepatic ablation in a porcine model: mitigation of body wall damage with a modified pulse sequence. Cardiovasc Intervent Radiol. 2019;42(7):1016–1023. 31041527 10.1007/s00270-019-02215-8 PMC7456499

[86] Lake AM, Xu Z, Wilkinson JE, Renal ablation by histotripsy-does it spare the collecting system? J Urol. 2008;179(3):1150–1154. 18206166 10.1016/j.juro.2007.10.033

[87] Vlaisavljevich E, Gerhardson T, Hall T, Effects of f-number on the histotripsy intrinsic threshold and cavitation bubble cloud behavior. Phys Med Biol. 2017;62(4):1269–1290. 27995900 10.1088/1361-6560/aa54c7 PMC5453715

[88] Bader KB. The influence of medium elasticity on the prediction of histotripsy-induced bubble expansion and erythrocyte viability. Phys Med Biol. 2018;63(9):095010. 29553049 10.1088/1361-6560/aab79b PMC5959013

[89] Woodacre JK, Landry TG, Brown JA. A low-cost miniature histotripsy transducer for precision tissue ablation. IEEE Trans Ultrason Ferroelectr Freq Control. 2018;65(11):2131–2140. 30222557 10.1109/TUFFC.2018.2869689

[90] Wang T-Y, Xu Z, Hall TL, An efficient treatment strategy for histotripsy by removing cavitation memory. Ultrasound Med Biol. 2012;38(5):753–766. 22402025 10.1016/j.ultrasmedbio.2012.01.013 PMC3462164

[91] Khokhlova TD, Canney MS, Khokhlova VA, Controlled tissue emulsification produced by high intensity focused ultrasound shock waves and millisecond boiling. J Acoust Soc Am. 2011;130(5):3498–3510. 22088025 10.1121/1.3626152 PMC3259668

[92] Khokhlova TD, Haider YA, Maxwell AD, Dependence of boiling histotripsy treatment efficiency on HIFU frequency and focal pressure levels. Ultrasound Med Biol. 2017;43(9):1975–1985. 28641910 10.1016/j.ultrasmedbio.2017.04.030 PMC5547902

[93] Simon JC, Sapozhnikov OA, Khokhlova VA, Ultrasonic atomization of tissue and its role in tissue fractionation by high intensity focused ultrasound. Phys Med Biol. 2012;57(23):8061–8078. 23159812 10.1088/0031-9155/57/23/8061 PMC3535451

[94] Simon JC, Sapozhnikov OA, Wang Y-N, Investigation into the mechanisms of tissue atomization by high-intensity focused ultrasound. Ultrasound Med Biol. 2015;41(5):1372–1385. 25662182 10.1016/j.ultrasmedbio.2014.12.022 PMC4398613

[95] Pahk KJ, de Andrade MO, Gelat P, Mechanical damage induced by the appearance of rectified bubble growth in a viscoelastic medium during boiling histotripsy exposure. Ultrason Sonochem. 2019;53:164–177. 30686603 10.1016/j.ultsonch.2019.01.001

[96] Maxwell AD, Yuldashev PV, Kreider W, A prototype therapy system for transcutaneous application of boiling histotripsy. IEEE Trans Ultrason Ferroelectr Freq Control. 2017;64(10):1542–1557. 28809681 10.1109/TUFFC.2017.2739649 PMC5871228

[97] Kim Y, Maxwell AD, Hall TL, Rapid prototyping fabrication of focused ultrasound transducers. IEEE Trans Ultrason Ferroelectr Freq Control. 2014;61(9):1559–1574. 25167156 10.1109/TUFFC.2014.3070

[98] Wang TY, Hall TL, Xu Z, Imaging feedback of histotripsy treatments using ultrasound shear wave elastography. IEEE Trans Ultrason Ferroelectr Freq Control. 2012;59(6):1167–1181. 22711412 10.1109/tuffc.2012.2307 PMC3746490

[99] Allen SP, Hernandez-Garcia L, Cain CA, MR-based detection of individual histotripsy bubble clouds formed in tissues and phantoms. Magn Reson Med. 2015;76(5):1486–1493. 26599823 10.1002/mrm.26062

[100] Lundt JE, Allen SP, Shi J, Non-invasive, rapid ablation of tissue volume using histotripsy. Ultrasound Med Biol. 2017;43(12):2834–2847. 28935135 10.1016/j.ultrasmedbio.2017.08.006 PMC5693635

[101] Kim Y, Vlaisavljevich E, Owens GE, *In vivo* transcostal histotripsy therapy without aberration correction. Phys Med Biol. 2014;59(11):2553–2568. 24785433 10.1088/0031-9155/59/11/2553

[102] Wang T-y, Xu Z, Winterroth F, Quantitative ultrasound backscatter for pulsed cavitational ultrasound therapy-histotripsy. IEEE Trans Ultrason Ferroelectr Freq Control. 2009;56(5):995–1005. 19750596 10.1109/tuffc.2009.1131 PMC3130252

[103] Wang TY, Hall TL, Xu Z, Imaging feedback for histotripsy by characterizing dynamics of acoustic radiation force impulse (ARFI)-induced shear waves excited in a treated volume. IEEE Trans Ultrason Ferroelectr Freq Control. 2014;61(7):1137–1151. 24960703 10.1109/TUFFC.2014.3013

[104] Allen SP, Vlaisavljevich E, Shi J, The response of MRI contrast parameters in in vitro tissues and tissue mimicking phantoms to fractionation by histotripsy. Phys Med Biol. 2017;62(17):7167–7180. 28741596 10.1088/1361-6560/aa81ed

[105] Worlikar T, Vlaisavljevich E, Gerhardson T, Histotripsy for Non-Invasive Ablation of Hepatocellular Carcinoma (HCC) tumor in a subcutaneous xenograft murine model. Annu Int Conf IEEE Eng Med Biol Soc. 2018;2018:6064–6067. 30441719 10.1109/EMBC.2018.8513650

[106] Vlaisavljevich E, Owens G, Lundt J, Non-invasive liver ablation using histotripsy: preclinical safety study in an *in vivo* porcine model. Ultrasound Med Biol. 2017;43(6):1237–1251. 28318889 10.1016/j.ultrasmedbio.2017.01.016

[107] Hall TL, Hempel CR, Wojno K, Histotripsy of the prostate: dose effects in a chronic canine model. Urology. 2009;74(4):932–937. 19628261 10.1016/j.urology.2009.03.049 PMC2757508

[108] Roberts WW, Teofilovic D, Jahnke RC, Histotripsy of the prostate using a commercial system in a canine model. J Urol. 2014;191(3):860–865. 24012583 10.1016/j.juro.2013.08.077

[109] Darnell SE, Hall TL, Tomlins SA, Histotripsy of the prostate in a canine model: characterization of post-therapy inflammation and fibrosis. J Endourol. 2015;29(7):810–815. 25566880 10.1089/end.2014.0585 PMC4507352

[110] Dubinsky TJ, Khokhlova TD, Khokhlova V, Histotripsy: the next generation of high-intensity focused ultrasound for focal prostate cancer therapy. J Ultrasound Med. 2020;39(6):1057–1067. 31830312 10.1002/jum.15191

[111] Schade GR, Styn NR, Ives KA, Prostate histotripsy: evaluation of prostatic urethral treatment parameters in a canine model. BJU Int. 2014;113(3):498–503. 24176120 10.1111/bju.12333 PMC3944657

[112] Schade GR, Styn NR, Hall TL, Endoscopic assessment and prediction of prostate urethral disintegration after histotripsy treatment in a canine model. J Endourol. 2012;26(2):183–189. 22050511 10.1089/end.2011.0349 PMC3311909

[113] Wheat JC, Hall TL, Hempel CR, Prostate histotripsy in an anticoagulated model. Urology. 2010;75(1):207–211. 19931897 10.1016/j.urology.2009.09.021 PMC2813892

[114] Pahk KJ, Shin C-H, Bae IY, Boiling Histotripsy-induced Partial Mechanical Ablation Modulates Tumour Microenvironment by Promoting Immunogenic Cell Death of Cancers. Sci Rep. 2019;9(1):9050. 31227775 10.1038/s41598-019-45542-z PMC6588624

[115] Hendricks A, Schmieley R, Howell J, Histotripsy initiates local and systemic immunological response and reduces tumor burden in breast cancer. American Association of Immunology Meeting; 2019 May 12, San Diego, CA.

[116] Hendricks A, Schmieley R, Howell J, Investigation of the local and systemic immune response to histotripsy ablation of breast cancer in a mouse model. Meeting of the International Society for Therapeutic Ultrasound; 2019 Jun 13–15, Barcelona, Catalonia.

[117] Hendricks-Wenger A, Zeher A, Sereno J, Histotripsy is an effective pancreatic tumor ablation strategy that releases immunostimulatory molecules and promotes anti-tumor immunity. 7th International Symposium on Focused Ultrasound. Virtual Meeting; 2020 Nov 8–13.

[118] Hendricks-Wenger A, Brock A, Gannon J, Determining the mechanism of the immune response to histotripsy ablation of pancreatic cancer. American Association of Immunologists Annual Meeting. J Immunol 204(1 Suppl.):241.2.

[119] Arnold L, Hendricks A, Coutermarsh-Ott S, Histotripsy treatment of primary osteosarcoma: feasibility study in excised canine tumors. 7th International Symposium on Focused Ultrasound. Virtual Meeting; 2020 Nov 8–13.

[120] Geldof AA, De Voogt HJ, Rao BR. High energy shock waves do not affect either primary tumor growth or metastasis of prostate carcinoma, R3327-MatLyLu. Urol Res. 1989;17(1):9–12. 2922893 10.1007/BF00261041

[121] Zhou L, Guo Y. *In vivo* effect of high energy shock waves on growth and metastasis of the heterografted tumors of nude mice. Chin Med J. 1996;109(2):157–161. 8758343

[122] Lafond M, Mestas J-L, Prieur F, Unseeded inertial cavitation for enhancing the delivery of chemotherapies: a safety study. Ultrasound Med Biol. 2016;42(1):220–231. 26478278 10.1016/j.ultrasmedbio.2015.08.019

[123] Oosterhof GO, Cornel EB, Smits GA, The influence of highenergy shock waves on the development of metastases. Ultrasound Med Biol. 1996;22(3):339–344. 8783466 10.1016/0301-5629(95)02051-9

[124] Miller DL, Dou C, Song J. Lithotripter shockwave-induced enhancement of mouse melanoma lung metastasis: dependence on cavitation nucleation. J Endourol. 2004;18(9):925–929. 15659934 10.1089/end.2004.18.925

[125] Tanter M, Thomas JL, Fink M. Focusing and steering through absorbing and aberrating layers: application to ultrasonic propagation through the skull. J Acoust Soc Am. 1998;103(5 Pt 1):2403–2410. 9604342 10.1121/1.422759

[126] Ammi AY, Mast TD, Huang I-H, Characterization of ultrasound propagation through *ex-vivo* human temporal bone. Ultrasound Med Biol. 2008;34(10):1578–1589. 18456391 10.1016/j.ultrasmedbio.2008.02.012 PMC4921610

[127] Elias WJ, Huss D, Voss T, A pilot study of focused ultrasound thalamotomy for essential tremor. N Engl J Med. 2013;369(7):640–648. 23944301 10.1056/NEJMoa1300962

[128] Chang WS, Jung HH, Kweon EJ, Unilateral magnetic resonance guided focused ultrasound thalamotomy for essential tremor: practices and clinicoradiological outcomes. J Neurol Neurosurg Psychiatry. 2015;86(3):257–264. 24876191 10.1136/jnnp-2014-307642

[129] Sukovich J, Xu Z, Kim Y, Targeted lesion generation through the skull without aberration correction using histotripsy. IEEE Trans Ultrason Ferroelectr Freq Control. 2016;63(5):671–682. 26890732 10.1109/TUFFC.2016.2531504 PMC7371448

[130] Gerhardson T, Sukovich JR, Pandey AS, Effect of frequency and focal spacing on transcranial histotripsy clot liquefaction, using electronic focal steering. Ultrasound Med Biol. 2017;43(10):2302–2317. 28716432 10.1016/j.ultrasmedbio.2017.06.010 PMC5580808

[131] Qureshi AI, Tuhrim S, Broderick JP, Spontaneous intracerebral hemorrhage. N Engl J Med. 2001;344(19):1450–1460. 11346811 10.1056/NEJM200105103441907

[132] Mendelow AD, Gregson BA, Fernandes HM, Early surgery versus initial conservative treatment in patients with spontaneous supratentorial intracerebral haematomas in the International Surgical Trial in Intracerebral Haemorrhage (STICH): a randomised trial. Lancet. 2005;365(9457):387–397. 15680453 10.1016/S0140-6736(05)17826-X

[133] Mendelow AD, Gregson BA, Rowan EN, Early surgery versus initial conservative treatment in patients with spontaneous supratentorial lobar intracerebral haematomas (STICH II): a randomised trial. Lancet. 2013;382(9890):397–408. 23726393 10.1016/S0140-6736(13)60986-1 PMC3906609

[134] Morgan T, Zuccarello M, Narayan R, Preliminary findings of the minimally-invasive surgery plus rtPA for intracerebral hemorrhage evacuation (MISTIE) clinical trial. Acta Neurochir Suppl. 2008;105:147–151. 19066101 10.1007/978-3-211-09469-3_30

[135] Nakamura T, Keep RF, Hua Y, Iron-induced oxidative brain injury after experimental intracerebral hemorrhage. Acta Neurochir Suppl. 2006;96:194–198. 16671453 10.1007/3-211-30714-1_42

[136] Xi G, Keep RF, Hoff JT. Mechanisms of brain injury after intracerebral haemorrhage. Lancet Neurol. 2006;5(1):53–63. 16361023 10.1016/S1474-4422(05)70283-0

[137] Gerhardson T, Sukovich JR, Pandey AS, Catheter hydrophone aberration correction for transcranial histotripsy treatment of intracerebral hemorrhage: proof-of-concept. IEEE Trans Ultrason Ferroelectr Freq Control. 2017;64(11):1684–1697. 28880166 10.1109/TUFFC.2017.2748050 PMC5681355

[138] Maxwell AD, Cain CA, Duryea AP, Noninvasive thrombolysis using pulsed ultrasound cavitation therapy – histotripsy. Ultrasound Med Biol. 2009;35(12):1982–1994. 19854563 10.1016/j.ultrasmedbio.2009.07.001 PMC2796469

[139] Goudot G, Khider L, Del Giudice C, Robotic assisted throm-botripsy allows high accurate venous recanalization in a porcine model of femoral venous thrombosis. Arch Cardiovasc Dis. 2019;11(1):100–101.

[140] Zhang X, Owens GE, Cain CA, Histotripsy thrombolysis on retracted clots. Ultrasound Med. Biol. 2016;42(8):1903–1918. 27166017 10.1016/j.ultrasmedbio.2016.03.027 PMC4912870

[141] Owens GE, Miller RM, Ensing G, Therapeutic ultrasound to noninvasively create intracardiac communications in an intact animal model. Catheter Cardiovasc Interv. 2011;77(4):580–588. 20853366 10.1002/ccd.22787 PMC3010446

[142] Kim Y, Gelehrter SK, Fifer CG, Non-invasive pulsed cavitational ultrasound for fetal tissue ablation: feasibility study in a fetal sheep model. Ultrasound Obstet Gynecol. 2011;37(4):450–457. 21433165 10.1002/uog.8880

[143] Kim Y, Fifer CG, Gelehrter SK, Developmental impact and lesion maturation of histotripsy-mediated non-invasive tissue ablation in a fetal sheep model. Ultrasound Med Biol. 2013;39(6):1047–1055. 23453378 10.1016/j.ultrasmedbio.2012.12.014

[144] Duryea AP, Roberts WW, Cain CA, Removal of residual cavitation nuclei to enhance histotripsy erosion of model urinary stones. IEEE Trans Ultrason Ferroelectr Freq Control. 2015;62(5):896–904. 25965682 10.1109/TUFFC.2015.7001 PMC4430129

[145] Osman MM, Alfano Y, Kamp S, 5-year-follow-up of patients with clinically insignificant residual fragments after extracorporeal shockwave lithotripsy. Eur. Urol 2005;47(6):860–864. 15925084 10.1016/j.eururo.2005.01.005

[146] Xu J, Bigelow TA, Halverson LJ, Minimization of treatment time for in vitro 1.1 MHz destruction of Pseudomonas aeruginosa biofilms by high-intensity focused ultrasound. Ultrasonics. 2012;52(5):668–675. 22341761 10.1016/j.ultras.2012.01.013

[147] Bigelow TA, Thomas CL, Wu H. Scan parameter optimization for histotripsy treatment of *S. Aureus* biofilms on surgical mesh. IEEE Trans Ultrason Ferroelectr Freq Control. 2020;67(2):341–349. 31634828 10.1109/TUFFC.2019.2948305 PMC7039400

[148] Bigelow TA, Thomas CL, Wu H, Histotripsy treatment of *S. Aureus* biofilms on surgical mesh samples under varying scan parameters. IEEE Trans Ultrason Ferroelectr Freq Control. 2018;65(6):1017–1024. 29856719 10.1109/TUFFC.2018.2819363 PMC6602080

[149] Childers C, Edsall C, Gannon J, Focused ultrasound biofilm ablation: investigation of histotripsy for the treatment of urinary catheter biofilms. 7th International Symposium on Focused Ultrasound. Virtual Meeting; 2020 NOV 8–13.

[150] Wang Y-N, Brayman A, Leotta D, Non-invasive treatment of abscesses by histotripsy. J Acoust Soc Am. 2019;146(4):2992–2992.

[151] Hingorani AP, Ascher E, Markevich N, Deep venous thrombosis after radiofrequency ablation of greater saphenous vein: a word of caution. J Vasc Surg. 2004;40(3):500–504. 15337880 10.1016/j.jvs.2004.04.032

[152] Chiang J, Cristescu M, Lee MH, Effects of microwave ablation on arterial and venous vasculature after treatment of hepatocellular carcinoma. Radiology. 2016;281(2):617–624. 27257951 10.1148/radiol.2016152508 PMC5084967

